# The RING finger protein family in health and disease

**DOI:** 10.1038/s41392-022-01152-2

**Published:** 2022-08-30

**Authors:** Chunmei Cai, Yan-Dong Tang, Jingbo Zhai, Chunfu Zheng

**Affiliations:** 1 Research Center for High Altitude Medicine, School of Medical, Qinghai University, Xi’ning, China; 2 State Key Laboratory of Veterinary Biotechnology, Harbin Veterinary Research Institute of Chinese Academy of Agricultural Sciences, Harbin, China; 3 Medical College, Inner Mongolia Minzu University, Tongliao, 028000 China; 4 Key Laboratory of Zoonose Prevention and Control at Universities of Inner Mongolia Autonomous Region, Tongliao, 028000 China; 5 The State Key Laboratory of Reproductive Regulation and Breeding of Grassland Livestock, School of Life Sciences, Inner Mongolia University, Hohhot, China; 6 Department of Immunology, School of Basic Medical Sciences, Fujian Medical University, Fuzhou, China; 7 Department of Microbiology, Immunology and Infectious Diseases, University of Calgary, Calgary, Alberta Canada

**Keywords:** Cancer, Innate immunity, Rheumatic diseases, Infectious diseases

## Abstract

Ubiquitination is a highly conserved and fundamental posttranslational modification (PTM) in all eukaryotes regulating thousands of proteins. The RING (really interesting new gene) finger (RNF) protein, containing the RING domain, exerts E3 ubiquitin ligase that mediates the covalent attachment of ubiquitin (Ub) to target proteins. Multiple reviews have summarized the critical roles of the tripartite-motif (TRIM) protein family, a subgroup of RNF proteins, in various diseases, including cancer, inflammatory, infectious, and neuropsychiatric disorders. Except for TRIMs, since numerous studies over the past decades have delineated that other RNF proteins also exert widespread involvement in several diseases, their importance should not be underestimated. This review summarizes the potential contribution of dysregulated RNF proteins, except for TRIMs, to the pathogenesis of some diseases, including cancer, autoimmune diseases, and neurodegenerative disorder. Since viral infection is broadly involved in the induction and development of those diseases, this manuscript also highlights the regulatory roles of RNF proteins, excluding TRIMs, in the antiviral immune responses. In addition, we further discuss the potential intervention strategies targeting other RNF proteins for the prevention and therapeutics of those human diseases.

## Introduction

Ubiquitination, a widespread posttranslational modification (PTM), plays a crucial role in spatially and temporally regulating the availability and activity of functional proteins, thus controlling many intracellular events.^1^ The ubiquitination mediates the covalent attachment of ubiquitin (Ub), a small, highly conserved, cytoplasmic protein of 76 amino acid residues, to target proteins involved in the various cellular processes.^2^ This process is achieved by the sequential actions of Ub-activating enzymes (E1), Ub-conjugating enzymes (E2), and Ub-ligating enzyme (E3): Briefly, the E1 relies on the energy released by ATP’s hydrolysis to activate the C‐terminal carboxyl of Ub and transfer the activated Ub onto the catalytic cysteine of an E2 to produce an E2~Ub conjugate (~ indicates a thioester bond). E3s then associate with the E2-Ub conjugate and target proteins to facilitate the formation of an isopeptide bond between the C‐terminal carboxyl of Ub and one of ubiquitin’s seven lysine residues (K6/11/27/29/33/48/63) or ubiquitin’s N-terminal methionine (M1) of substrate.^3–5^ E3 ubiquitin ligase links the mono‐ or poly- Ub linkage types to determine the chain topology and form the basis for the pleiotropic cellular functions of Ub chains.^6^ For example, K48-linked Ub chains are the canonical signal to the target protein for proteasomal degradation, whereas K63- and M1-based Ub chains generally are nonproteolytic signals and regulate signal transduction, modulation of innate immunity, intracellular trafficking, and DNA damage response.^7–10^ Those not linked via canonical K48 or K63 linkages are atypical Ub chains, whose cellular function or regulation remains less known.^3,11^

The RNF family, containing the N-terminal RING domain, is the largest E3 ubiquitin ligase family with 340 validated human members.^12^ The RING domain was first predicted to have a role in DNA binding and recognition.^13^ Bailly et al. found that yeast DNA-damage repair protein Rad18, a member of the original cohort of RNF proteins, can mediate histone ubiquitination related to its RING domain.^14^ Since then, many studies have focused on the relationship between the RING domain and ubiquitination modification. When combined with two zinc atoms, the RING domain forms a RING finger structure similar to the zinc finger, which can form a relatively stable binding region to provide a structural basis for the combination of E2 and to mediate the ubiquitination process effectively.^15^ Emerging evidence has shown that the RNF proteins are involved in many biological processes, and the abnormal function of RNF proteins, caused by genetic alterations, frequently contributes to several diseases, classified as cancer, immunological disease, and neuropsychiatric disorders. Besides, viral infection is a leading cause of infection-related cancers, accounting for over 15% of all cancers.^16–18^ It is also proposed to induce or exacerbate autoimmune responses or protect from certain immune disorders^17,19^ and contribute to the pathogenesis of neurological disorders.^20,21^ Broad implications revealed the critical role of RNF proteins in the control or pathogenesis of viral infection, which remains the most formidable challenge to humankind, especially the pandemic coronavirus disease 2019 (COVID‐19) caused by severe acute respiratory syndrome coronavirus 2 (SARS‐CoV‐2). Elucidating the regulatory roles of RNF proteins may not only provide the potential intervention strategies for the prevention and therapeutics of viral infection but also provide new insights into developing novel and effective therapeutic strategies for the above human diseases.

However, previous studies primarily aimed at the function of the tripartite-motif (TRIM) protein family, a subfamily of RNF proteins, while underestimated the importance of other RNF proteins in the therapy and/or pathogenesis of human diseases. Here, we detail the association of dysregulated RNF proteins, excluding TRIMs, with various diseases, including cancer, inflammatory diseases, and neurological disorders. We also focus on the mechanisms other RNF proteins adopted to regulate antiviral host responses to affect the control or pathogenesis of viral infection. Moreover, this review summarizes and discusses the potential role of other RNF proteins as a novel biomarker or therapeutic target.

## The classification, structure, and function of RNF family proteins

**Fig. 1:**
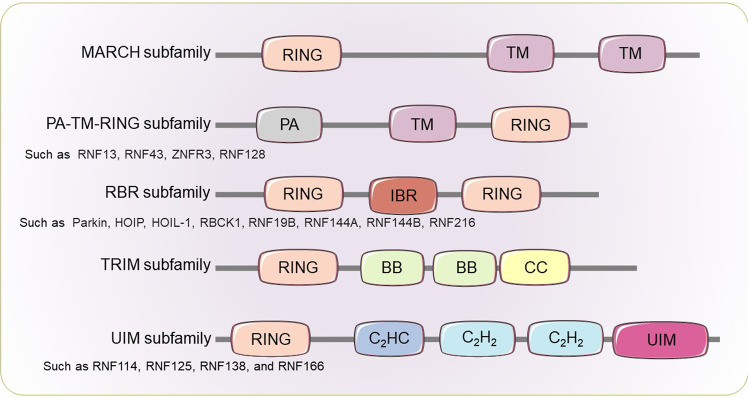
Classification of the RING finger (RNF) family. All members of the RNF family are characterized by the N-terminal RING domain. Each subfamily has its unique domains besides the conserved RING domain. The MARCH subfamily is characterized by the TM domain. The PA-TM-RING subfamily is characterized by the PA domain and two TM domains. The RBR subfamily is characterized by the IBR domain. The TRIM subfamily is characterized by two BB domains and a CC domain. The UIM subfamily is characterized by a C2HC-type zinc finger, two C2H2-type zinc fingers, and UIM

The RNF family proteins are conserved from yeast to humans, with more than 600 E3s estimated to be encoded by the mammalian genome, surpassing the 518 protein kinase genes.^22^ The RNF proteins are broadly classified into five subfamilies that share a common N-terminal RING domain with various unique domains (Fig. 1).^23^ The RING domain is shown to confer E3 activity, which allows RNF proteins to conjunct the Ub, small ubiquitin-like modifier (SUMO), or ubiquitin-like molecule IFN-stimulated protein of 15 kDa (ISG15), loaded by E2, to a wide variety of substrates, contributing to the biological flexibility of RNF proteins.

The Membrane-associated RING-cysteine-histidine (CH) (MARCH) family consists of 11 mammalian members that harbor one C4HC3 cysteine-histidine (RING-CH) domain and multiple transmembrane (TM) domains.^24^ Except for MARCH7 and MARCH10, the other 9 members share a similar structure: an N-terminal RING-CH domain, followed by two or more C-terminal TM domains. The MARCH7 and MARCH10 have no TM domain, with their RING-CH domain at the C-terminus. Most MARCH proteins are abundantly expressed in immune cells, such as monocytes, macrophages, dendritic cells, B cells, and T cells, suggesting the potential to regulate immune responses. The MARCH proteins are involved in immunity, neurodegenerative disease, cancer, autoimmunity, and viral infection by regulating cell-surface antigen-presenting proteins, immune receptors, tumor immune checkpoints, components in innate immunity, or viral proteins.^24–26^

The PA-TM-RING family (11 members in humans) is characterized by three conserved domains, including a protease-associated (PA) domain, a TM domain, and a RING-H2 domain.^27^ The PA domain is responsible for protein-interacting. These endosomal membrane proteins appear short-lived and expressed at low levels in mammals.^28^ Few reports have demonstrated the physiological roles of PA-TM-RING proteins, which involve cellular endosome trafficking, cell proliferation, autoimmunity, cancer, and antiviral immunity.^27,28^

The RING between RING (RBR) family encompasses 14 distinct human enzymes that are defined by two RING domains (RING1 and RING2) connected via an In-Between-RING (IBR) domain.^29^ Similar to canonical RINGs, the RBR RING1 domain interacts with E2~Ub in a RING E3-like fashion, promoting the transfer of Ub to a conserved catalytic Cys in the RING2 domain to form a reactive HECT-like E3∼Ub thioester intermediate. The covalent E3~Ub intermediate facilitates the transfer of ubiquitin to its target protein. However, the exact function of the IBR domain remains enigmatic. The remarkable functional diversity of a few RBR proteins has been identified, including regulation of transcription, RNA metabolism, translation, subcellular tethering, and the activity and stability of proteins involved in cellular signaling, stress signaling, and cell-cycle.^29,30^ Therefore, the dysfunction of several RBR proteins is implicated in the pathogenesis of neurodegenerative disease, cancer, autoimmunity, and viral infection.

The TRIM superfamily exhibits a highly conserved order of domains in the RBCC motif, comprising a RING domain, one or two B-box (BB) domains, and a coiled-coil (CC) domain.^31^ The BB domains of TRIMs are also zinc-binding motifs, whereas the function of BB domains remains less so far known. The CC domain mediates homomeric and heteromeric assemblies of TRIMs and other proteins, which is pivotal for TRIMs’ activity. In addition, the C-terminal domains of TRIMs generally serve as a scaffold for recruiting different sets of corresponding proteins while also display enzymatic activity or bind nucleic acids. The TRIM proteins have emerged as potent regulators in many biological processes, including autophagy, cancers, genetic disorders, neurological disorders, and immunity, especially antiviral immune responses.^23,31,32^

The RING-Ub interacting motif (UIM) (RING-UIM) family only consists of 4 members (RNF114, RNF125, RNF138, and RNF166) that harbor a RING domain, a C_2_HC and two C_2_H_2_ type zinc fingers, as well as a UIM-type domain.^23,33^ However, the exact function of each domain (except the RING domain) remains enigmatic. A report delineated that the UIM and RING domains of RNF125 are required for its autoubiquitination, which appears to control the RNF125 stability.^33^ Few studies have indicated the roles of RING-UIM proteins that positively regulate T cell activation (RNF138 is elusive) and virus-triggered IFN responses (all RING-UIM members).^23,33^

## RNF family proteins in diseases

### Cancer

Cancer is a multifactorial disease considered a major public health problem worldwide.^34^ Given the crucial role of RNF proteins-mediated ubiquitination, involved in almost every cellular process, it is not surprising that their dysregulation caused by genetic alterations is associated with the development, progression, and response to therapy of human cancers (Table 1).^35,36^ However, few studies have addressed whether and how certain mutations in RNF proteins contribute to tumorigenesis.

**Table 1:** Reported RNF Proteins Mutations in Human Patients

Gene name	Gene alteration	Disease	Reference
Parkin	E344G, R275Q, T173A, R42C, and I2V (heterozygous)	Glioblastoma	^39^
Parkin	Copy number loss (heterozygous)	Glioblastoma	^39^
MDM2	Amplification of MDM2 in 7.1% of glioblastoma	Glioblastoma	^41^
ZNRF3	p.Q167, p.E173, p.Q198, p.C233, p.V320M, p.N392fs, and p.P445L (homozygous)	Adrenocortical carcinoma	^46^
ZNRF3	Copy number loss (homozygous)	Adrenocortical carcinoma	^46,47^
Parkin	N254S, D243N, and H279P (heterozygous) and A46T(homozygous)	Lung cancer	^39^
Parkin	p.R275W (heterozygous)	Lung cancer	^49^
RNF55	S80N/H94Y, Q249E, and W802* (heterozygous)	Lung cancer	^52^
RNF55	Copy number loss (heterozygous)	Lung cancer	^52^
Skp2	Amplification of *Skp2* gene	Lung cancer	^53,54^
Parkin	Copy number loss	Breast, serous ovarian, and bladder cancers	^50^
Parkin	Promoter methylation of *Parkin* gene	Breast cancer	^57^
MDM2	Amplification of MDM2 in 7% (40/661) of breast cancer	Breast cancer	^58^
RNF43	10 different RNF43 somatin mutations (heterozygous)	Ovarian cancer	^61,62^
RNF43	p.G659fs and p.R117fs	Endometrial cancer and colorectal cancer	^65^
RNF43	16 different RNF43 somatin mutations (heterozygous)	Gastric cancer	^69^
RNF180	Promoter methylation of *RNF180* gene	Gastric cancer	^70–72^
MDM2	Amplification of MDM2 in gastric cancer	Gastric cancer	^73,74^
RNF43	5 frameshift mutations (p.F69fs, p.S264fs, p.L311fs, p.R363fs, and p.V490fs), 1 non-sense mutation (p.Q153X), and 2 missense mutations (p.I164N and p.P310A)	Pancreatic cancer	^78^
Parkin	Copy number loss (heterozygous), promoter methylation of *Parkin*, and p.C211T mutation	Colorectal cancer	^85^
MDM2	Amplification of MDM2 in 9-18% of colorectal cancer	Colorectal cancer	^86,87^
RNF55	16 different RNF55 mutations (homozygous)	MDS/ MPN	^89^
RNF55	Over 50 different RNF55 mutations in JMML (homozygous)	juvenile myelomonocytic leukemia	^92–94^
Parkin	Promoter methylation of *Parkin* gene	Acute lymphoblastic leukemia and chronic myeloid leukemia	^98^
MDM2	Amplification of MDM2 in B-cell chronic lymphocytic leukemia	B-cell chronic lymphocytic leukemia	^99^
BIRC2/ BIRC3	Copy number loss (homozygous)	Multiple myeloma	^101^
BIRC3	Over 20 different BIRC3 mutations in MCL (heterozygous)	Mantle cell lymphoma	^102,103^
BIRC3	6 different mutations and copy number loss in SMZL (heterozygous)	Splenic marginal zone lymphoma	^104^
RNF31	p.Q584H and p.Q622L	Activated B cell-like (ABC) subtype of diffuse large B cell lymphoma (DLBCL)	^105^
RNF56	rs3772534 A/G	Systemic lupus erythematosus	^118^
RNF56	rs9657904 T/C	Multiple sclerosis	^121^
RNF56	rs3772534 A/G	Type 1 diabetes in children	^129^
RNF31	L72P (homozygous)	multi-organ auto-inflammation, combined immunodeficiency, subclinical amylopectinosis, and systemic lymphangiectasia	^132^
RNF54	L41fsX7 (homozygous) and Q185X;c.ex1_ex4del (compound heterozygous)	chronic auto-inflammation, invasive bacterial infections, and muscular amylopectinosis	^133^
RNF216	p.R717C (heterozygous)	A syndrome of cerebellar ataxia, dementia, and hypogonadotropic hypogonadism	^179^
RNF220	p.R363Q and p.R365Q (homozygous)	progressive ataxia and deafness (AR-LAD)	^180^
Parkin	Over 100 different Parkin mutations affecting each of Parkin’s 12 exons	Parkinson’s disease	^138,139^

#### Glioma

Glioma is the most common malignant primary tumor of the central nervous system with varying malignancy grades I-IV and histological subtypes, including astrocytomas, glioblastoma multiform (GBM), oligodendrogliomas, and mixed tumors.^37^ GBM is the most lethal glioma with a poor prognosis for their resistance to conventional therapy and easily recrudescent, accounting for 70% of all diffuse glioma diagnoses.^38^ Chromosomal microarray analysis has shown Parkin’s genetic alteration (including mutations and copy number loss) in glioblastoma and lung cancer.^39^ As a potential tumor suppressor gene at chromosome 6q25-q27, both *Parkin* mutations and deletions could be risk factors for glioma.^40^ Mutational inactivation of Parkin may abolish its ability to block both tumor cell growth and to ubiquitinate and degrade cyclin E, thus loss-of-function suppressing tumorigenicity. The array-based comparative genomic hybridization revealed that murine double minute 2 (MDM2) overexpression, a RING finger E3 ubiquitin ligase, is observed in glioblastoma, which is caused by amplification of the *MDM2* gene, located on chromosome 12 (12q14–15).^41^ Since the MDM2 can reduce the level and activity of p53, one of the most important tumor suppressors, the mutational inactivation of MDM2 could contribute to glioblastoma development. Of note, nutlins, a new class of small molecules, physically interact with MDM2 to prevent its association with p53, consequently enhancing p53 activity and p53-dependent apoptotic pathway for inhibiting the growth of glioblastomas.^42,43^

#### Adrenocortical Carcinoma

Adrenocortical carcinoma (ACC) is a rare and aggressive malignancy typically poor prognosis.^44^ ACC is capable of secreting excess adrenocortical hormones, thus compounding morbidity and compromising clinical outcomes.^45^ An exome sequencing and SNP array analysis in 121 ACCs showed the recurrent homozygous deletions and mutations of *RNF203* (*ZNRF3*), encoding a cell surface E3 ubiquitin ligase, which is located at 22q12.1.^46^ The *RNF203* is the most frequently altered gene (21%) and may be a novel tumor suppressor gene that acts as a negative feedback regulator of WNT/β-catenin signaling. Another study performed whole-exome sequencing also indicated the homozygous deletion of WNT repressors *ZNRF3* (4/41 9.8%) in ACC cases.^47^

#### Lung Cancer

Lung cancer is one of the most commonly diagnosed cancers and the most prominent cause of cancer-related mortality worldwide.^48^ Lung cancer is broadly divided into two major histological types, non-small-cell lung cancer (NSCLC) (85% of total diagnoses) and small-cell lung cancer (SCLC) (15% of total diagnoses). As mentioned in glioma, the *Parkin* somatic mutations and intragenic deletions also have been linked to lung cancer and further identified the ubiquitin E3 ligase Parkin as a tumor suppressor in lung cancer.^39,40^ Of note, whole-exome sequencing identified a germline mutation in *Parkin*, p.R275W, located in the highly conserved and functionally important RING 1 domain of Parkin.^49^ This rare mutation causes subcellular mislocalization of Parkin and impairs Parkin-mediated degradation of cyclin E, thereby accelerating cell-cycle progression for lung cancer tumorigenesis.^49–51^ The *RNF55* (*c-Cbl*), located at chromosome 11, is frequently mutated or even lost in lung cancers, and all genetic alterations are heterozygous.^52^ The overall mutations of *RNF55* remarkably promote cellular viability and motility, contributing to the NSCLC tumorigenesis and metastasis. In contrast, the *Skp2*, located at chromosome 5p13, which is commonly overrepresented in lung cancer, is amplified in 7 (44%) of 16 primary SCLCs and 106 (65%) of 163 NSCLCs, consequently overexpressed.^53,54^ The amplification of *Skp2*, encoding Skp2 that is putatively involved in regulating cell cycle progression by controlling the degradation of p27^*kip1*^, p21^*cip1*^, p57^*kip2*^, p130-Rb2, and cyclin E, may play a crucial role in carcinogenesis and development of lung cancer. Of note, the *Skp2* overexpression in NSCLCs with *RAS* mutation is an independent poor prognostic marker.^53^

#### Breast cancer

Breast cancer remains the leading cancer-related cause of death from cancer in women worldwide.^55^ Breast cancer is a complex disease, showing a large degree of inter and intra-tumoral heterogeneity. Clinically, specific subtypes of this cancer are based primarily on their histopathological appearance and expression of estrogen receptor (ER), progesterone receptor (PR), and ERBB2 receptor (HER2).^56^ A pan-cancer analysis revealed that *Parkin* is frequently deleted in breast cancer (32% deletion rate).^50^ Cancer-specific alteration of Parkin abrogates its tumor-suppressive function for the involvement of this E3 Ub ligase in proteasomal degradation of cyclin D and cyclin E, which subsequently control cell cycle progression. In addition, Khushnuma *et al*. reported that aberrant promoter methylation of *Parkin* is a frequent incident, which causes its epigenetic inactivation in breast cancer via reducing the expression of Parkin protein.^57^ Parkin’s methylation and copy number loss have been linked with poor prognosis in breast cancer patients. The increased *MDM2* expression, caused primarily by gene amplification, is also observed in breast cancer, contributing to tumorigenicity via suppressing p53 function.^58^ They further implicated that the *MDM2* amplification may serve as an adverse prognostic parameter only in ER^+^ early-stage breast cancer.

#### Ovarian and endometrial cancers

The tumors of the female genital tract, including ovarian, endometrial, and cervical cancers, represent a leading cause of cancer-related morbidity and mortality in women worldwide.^59^ Ovarian cancer, an aggressive epithelial tumor, represents a leading cause of cancer-related morbidity and mortality in women.^60^ Main histological types of ovarian cancers include epithelial tumors, germ cell tumors, sex cord-stromal cell tumors, and metastatic tumors. Epithelial cancers are the most common, accounting for 90% of all cases, in which subtype the most prevalent is serous ovarian carcinoma (52%). *Parkin* deletion also occurs in ovarian carcinoma with a 62% rate, abrogating its ability to suppress tumor growth.^50^ In addition, the whole-exome sequencing data revealed that the *RNF43* is frequently mutated in mucinous ovarian carcinomas: mutation in 13.3% (2/15) or 21% (6/29) mucinous ovarian carcinomas and 9% (2/22) mucinous ovarian borderline tumors.^61,62^ Since the RNF43 can block WNT signaling by selectively targeting the Frizzled receptor for ubiquitin-mediated degradation, inactivating mutations of *RNF43* may contribute to the development of ovarian mucinous tumors.^61–63^

Endometrial cancer (EC), originating from the uterine epithelium, is rising in incidence and related mortality among women of all backgrounds.^64^ The ECs can be divided into histological subtypes, including endometrioid, serous, clear cell and mixed ECs, and uterine carcinosarcoma [G]. The endometrioid EC is generally more clinically aggressive. The whole-exome sequencing data revealed that the somatic gene *RNF43*, encoding a tumor suppressor targeting the WNT pathway, is mutated in 18-27% of endometrioid ECs.^65,66^ Most of those genetic alterations are frameshift mutations, which are predicted to cause loss of function of RNF43 on suppressing the tumorigenicity of EC.

#### Gastric Cancer

Gastric cancer is a heterogeneous disease representing the second most common cause of cancer death worldwide.^67^ Gastric cancer is classified broadly as cardia gastric cancer (arising in the area of the stomach adjoining the esophagogastric junction) and non-cardia gastric cancer (arising from more distal regions of the stomach).^68^ An integrative genomic analysis reported the somatin mutation of *RNF43*, encoding a negative regulator of the WNT pathway, in 4.8% of microsatellite stable (MSS) and 54.6% of microsatellite instable (MSI) group of gastric cancers.^69^ Mutational inactivation of *RNF43* may contribute to deregulated WNT activity, thus loss-of-function suppressing gastric cancer progression. Genome-wide methylation screening among 198 gastric tumors revealed that the promoter methylation of *RNF180* is detected in 76% of cases but none of the normal controls, indicating the RNF180 transcript is commonly silenced in gastric cancer.^70^ They further demonstrated that the RNF180 is a novel potential tumor suppressor in gastric cancer by suppressing cell growth and inducing apoptosis. Besides, additional studies further suggested that the methylation of key CpG sites or hypermethylated CpG site count of *RNF180* DNA promoter may predict the prognosis of gastric cancer patients in clinics.^71,72^ The *MDM2* gene amplification is often observed in gastric cancer, potentially also contributing to this cancer tumorigenesis.^73,74^ As the tumors with *MDM2* gene amplification respond well to MDM2 antagonists,^42,75^ MDM2 is a promising target for gastric cancer therapy.

#### Pancreatic cancer

Pancreatic cancer is expected to continue to represent a leading cause of cancer-related death and has poor survival rates, which have not improved over the past few decades.^76,77^ Pancreatic ductal adenocarcinoma (PDAC) constitutes 90% of pancreatic cancers and poses a major medical problem. A whole-exome sequencing reported the somatin mutations of *RNF43*, targeting the frizzled receptor for its ubiquitination and degradation to block WNT signaling activation, in 14% (8/57) of frozen intraductal papillary mucinous neoplasms of the pancreas.^78^ In addition, they first revealed the significant association between *RNF43* mutation in frozen samples and downregulated *RNF43* expression in 52 (29.5%) of the 176 surgically resected cases, suggesting the reduced expression of *RNF43* may result from its mutations. However, the reduced *RNF43* expression is not associated with any clinicopathological features. Several studies also delineated the *RNF43* mutations in intraductal papillary mucinous neoplasms.^79–81^ Another exome sequencing of genomic DNA, extracted from blood and the cancer biopsy from a patient with stage IV metastatic pancreatic cancer, showed a splice site mutation in *RBCK1* (*RNF54* or *HOIL-1*), which genetic alteration may be the most promising driver of pancreatic cancer.^82^

#### Colorectal cancer

Colorectal cancer (CRC) is one of the lethal cancers, accounting for approximately 10% of all cancer incidence and cancer-related mortality worldwide.^83^ Based on gene expression, this disease is divided into four consensus molecular subtypes: CMS1 features hypermutation, unstable microsatellite, and strong immune activation; CMS2 presents with epithelial features and marked WNT and MYC signaling activation; CMS3 exhibits epithelial features and evident metabolic dysregulation; as well as CMS4 possesses prominent TGF-β activation, stromal invasion and angiogenesis.^84^ Whole-exome sequencing identified the somatic mutation of *RNF43* in 18.9% (35/185) of CRCs.^65^ Most *RNF43* mutations are truncating events (defined as frameshift indels, nonsense mutations, and splice-site mutations), leading to loss of function of RNF43 in suppressing the tumorigenicity of CRC for enhanced WNT pathway activation. An array of comparative genomic hybridization showed the *Parkin* deletion in 33% (33/100) CRCs, and the *Parkin* expression levels are dramatically lower in the CRC samples with deleted *Parkin* than in wild-type *Parkin.*^85^ In human CRC, the heterozygotic *Parkin* deletion is significantly associated with adenomatous polyposis coli (*APC*) deficiency, which plays an important role in regulating WNT signaling. The heterozygotic *Parkin* deletion cooperates with *APC* suppression to accelerate CRC progression, while this cooperation’s underlying mechanism remains enigmatic.

Moreover, the *Parkin* mutations and promoter hypermethylation are also observed in CRC, which genetic alterations are predicted to cause the loss of function of Parkin in suppressing CRC tumorigenesis. The *MDM2* gene amplification and increased expression are detected in 9-18% of the human CRCs, indicating the oncogenic role of MDM2 by promoting the phosphorylated degradation of p53 to block this transcriptional factor activity.^86,87^ Besides, the frequency of *MDM2* gene amplification is remarkably correlated to tumor stage, implicating the *MDM2* gene amplification may contribute to CRC progression at late rather than early.^86^ Since the tumors with *MDM2* gene amplification respond well to MDM2 antagonists,^42,75^ these small-molecule MDM2 antagonists may offer promising agents for cancer therapy in CRC.

#### Myeloid and lymphoid neoplasms

Myeloid malignancies are broadly classified into three categories: myelodysplastic syndromes (MDS)/ myeloproliferative neoplasms (MPN) associated with ineffective hematopoiesis, acute myelogenous leukemia (AML) featured by an accumulation of immature myeloid cells in the bone marrow, and chronic myeloproliferative disorders (MPDs) generally characterized by increased production of terminally differentiated myeloid cells.^88^ The *Cbl* (*c-Cbl* or *RNF55*), encoding an E3 ubiquitin ligase that suppresses the JAK-STAT signaling via targeting the tyrosine kinase receptor for ubiquitin-mediated degradation, is mutated in roughly 5% of 222 patients with MDS/ MPN.^89^ The homozygous *Cbl* (*c-Cbl* or *RNF55*) mutations frequently inhibit the E3 ubiquitin ligase activity of its wild-type gene product and homologous Cbl-b (RNF56), leading to excessive sensitivity to a variety of growth factors to induce constitutive activation of JAK-STAT signaling, which consequently contributes to the pathogenesis of MDS/ MPN. Moreover, these *Cbl* mutations are correlated with the poor prognosis of MDS/MPN patients.^90^

Juvenile myelomonocytic leukemia (JMML) is an aggressive MPN of early childhood characterized by hyperactivation of the RAS signal transduction pathway.^91^ The *Cbl* (*c-Cbl* or *RNF55*), encoding an RNF E3 ubiquitin ligase that mediates the decay of receptor tyrosine kinases in the RAS pathway, is homozygously mutated in approximately 10-15% of patients with *de novo* JMML.^92,93^ The mutational inactivation of Cbl causes the hyperactive RAS signaling, consequently enhancing the carcinogenesis of JMML.^92,93^ Besides, the *Cbl* family mutations are significantly correlated with the development and poor prognosis of JMML patients.^92^ Moreover, children with germline *Cbl* mutations are at increased risk of developing JMML, and patients with *Cbl*‐mutated JMML are self‐limiting with splenomegaly decreasing over the years.^94^ Another MPN is chronic myelomonocytic leukemia (CMML), a hematologic malignancy exclusively affecting the elderly.^95^ A whole-exome sequencing screen identified the *ARIH1*, encoding an RBR E3 ubiquitin ligase, is frequently mutated in 21 CMML patients, while the expression levels do not differ between CD34^+^ or CD14^+^ cells from CMML samples, implicating the ARIH1 may not contribute to CMML pathogenesis.^96^ Chronic myeloid leukemia (CML) also belongs to the MPN group.^97^ A fluorescence in situ hybridization (FISH) study identified that the *Parkin* promoter is aberrantly methylated in 20% of 60 patients with CML and 26% of 195 patients with *de novo* acute lymphoblastic leukemia (ALL),^98^ a predominantly disease of childhood. In addition, the reduced expression of *Parkin* is closely related to its promoter’s hypermethylation, thus the abnormal methylation of the *Parkin* promoter potentially contributes to the pathogenesis of CML and ALL. The *MDM2* amplification is also observed in 56% of 60 patients with B-cell CLL, leading to overexpression of *MDM2*, which consequently contributes to tumorigenicity of CLL via suppressing the activation of p53 signaling.^99^

Multiple myeloma is a malignancy of terminally differentiated plasma cells and is typically characterized by the secretion of a monoclonal immunoglobulin protein in the serum and/or urine.^100^ An integrated analysis of high-resolution aCGH and GEP identified the homozygous deletions of *BIRC2*/ *BIRC3* (*RNF48*/ *RNF49*), tumor suppressor genes involved in suppressing the activation of non-canonical NF-κB signaling pathways.^101^ The inactivation of *BIRC2*/ *BIRC3* by bi-allelic deletion is correlated with the constitutive activation of the non-canonical NF-κB signaling observed in approximately 20% of patients with multiple myeloma, suggesting the *BIRC2*/ *BIRC3* genetic alterations potentially contribute to the pathogenesis of multiple myeloma. The *BIRC3* (*RNF49*) is also frequently mutated in 6–10% of patients with mantle cell lymphoma (MCL), a mature CD5^+^ B-cell neoplasm.^102,103^ The loss-of-function mutations in *BIRC3* release NIK from the inhibition, which results in NIK-NF-κB activation, consequently contributing to the aggressive behavior of MCL. The genetic alterations of *BIRC3* are also observed in 11% of 101 patients with splenic marginal zone lymphoma (SMZL), a small B-cell lymphoma.^104^ All somatic alterations, including 6 mutations and 5 deletions of the *BIRC3* gene, are predicted to eliminate or truncate the E3 ubiquitin ligase activity of BIRC3, which blocks its inhibitory function on non-canonical NF-κB pathway activation, contributing to the development of SMZL. In addition, the germline mutations in *HOIP* (*RNF31*), encoding one component of the linear ubiquitin chain assembly complex (LUBAC), which mediated linear poly-ubiquitination is essential for activation of canonical NF-κB signaling pathway, are observed in 7.8% of patients with activated B cell-like (ABC) subtype of diffuse large B-cell lymphoma (DLBCL).^105^ These two alterations, p.Q584H and p.Q622L, enhance the HOIP-HOIL-1 association, facilitating NF-κB activity in ABC DLBCL. Moreover, targeting the HOIP-HOIL-1 interface with a synthesized peptide based on the p.Q622L polymorphism significantly decreases the NF-κB activation and increases the death of ABC DLBCL cells implicating the potential therapeutic target of LUBAC in ABC DLBCL.

### Autoimmune diseases

Autoimmune diseases often involve dysregulated innate and adaptive immunity against anatomical self-antigens, in which overactive immune responses attack self-tissues.^106,107^ According to the extent of tissues involved, these diseases are divided into organ-specific (such as type I diabetes (T1D), multiple sclerosis (MS), and inflammatory bowel diseases (IBDs)) and systemic (such as systemic lupus erythematosus (SLE) and rheumatoid arthritis (RA)). The RNF family proteins-mediated ubiquitination may be a major player in autoimmunity for genetic mutations (Table 1).

#### Rheumatoid arthritis (RA)

RA is a chronic inflammation of joints and other associated tissues with varying systemic involvement in the presence of rheumatoid serum factor and anti-cyclic citrullinated peptide antibodies.^108^ The expression of RNF85 (TRAF6) is upregulated in fibroblast-like synoviocytes (FLS) of RA patients.^109^ The enhanced RNF85 expression in RA-FLS results in induced high expression of vascular cell adhesion molecule-1 (VCAM-1) and immune cell infiltration of the synovium via regulating the activation of NF-κB and MAPKs/AP-1, consequently contributing to the progression of RA.^110,111^ In addition, more recent work revealed that the ubiquitin-like containing PHD and RING finger domains 1 (UHRF1, also named RNF106) acts as a central epigenetic regulator to suppress gene expression of multiple exacerbating factors in RA progression through maintaining the DNA methylation status during DNA replication.^112^ The expression levels of RNF106 significantly increase in synovial fibroblasts (SFs) from arthritis model mice and RA patients. The SF-specific *Uhrf1* conditional knockout mice exhibit more severe arthritic phenotypes than controls. They further identified Ryuvidine as a candidate chemical to stabilize RNF106 to improve disease features in a mouse model of RA.

#### Systemic lupus erythematosus (SLE)

SLE is a systemic autoimmune disease that primarily affects women of gestational age and damages various tissues and organs.^113^ This disease is characterized by polyreactive autoantibodies that bind to different host targets, including proteins, nucleic acids, and their complexes. Abnormalities in the function of T and B lymphocytes and the signaling pathways induced through their receptors play a crucial role in the pathogenesis of SLE.^114–117^ Mice with B cell-specific *RNF55* (*c-Cbl*) and *RNF56* (*Cbl-b*) deficiency manifest spontaneously SLE-like disease.^115^ Cbl-dko B cells are not hyperactive in terms of proliferation and antibody production upon BCR stimulation both in vivo and in vitro. Notably, impaired B-cell anergy to self-antigen is observed in B cell-specific Cbl-dko mice, leading to impaired B-cell tolerance, which may be one of the major reasons contributing to the pathogenesis of the SLE-like autoimmune disease. Besides, a significant association between the 2126(A/G) SNP in exon 12 of the *Cbl-b* gene and SLE was detected in the Mexican mestizo population, implicating the potential contribution of this mutation in SLE pathogenesis.^118^

The RNF84 (TRAF5) stabilizes the RAR-related orphan receptor-γt (RORγt) via catalyzing K63-linked ubiquitination, thus facilitating the proinflammatory Th17 cell differentiation and IL17A/IL17F expression to aggravate inflammatory responses.^116^ Consistent with this observation, elevated expression of RNF84 is positively correlated with IL-17A in CD4^+^ T-cells from SLE patients, implicating that the RNF84 may contribute to SLE pathogenesis by ubiquitinating RORγt under inflammatory conditions. Recent work denoted that RNF106 (UHRF1) is downregulated in circulating T follicular helper (Tfh) cells isolated from peripheral blood mononuclear cells of SLE patients.^117^ They further showed that the RNF106 inhibits the expression of B cell lymphoma 6 (BCL6) by decreasing DNA methylation and H3K27me3 levels, thus enhancing Tfh cell differentiation, which cell group may be involved in the dysregulated antibody responses associated with SLE. The field requires further data to determine how RNF106 modulates the BCL6 transcription in Tfh cells of SLE.

#### Multiple sclerosis (MS)

MS is the most prevalent chronic inflammatory disease of the central nervous system characterized by inflammatory demyelination with axonal transection.^119,120^ This disorder is triggered by environmental factors that act on a genetically susceptible host. A genome‐wide association study (GWAS) showed a significant association of *RNF56* (*Cbl‐b*) gene variants with MS, confirmed in 1775 cases and 2005 controls.^121^ In addition, mice lacking the Cbl‐b ortholog are prone to develop experimental autoimmune encephalomyelitis (EAE), the animal model for MS, for hyperactive Th17 cell responses, thereby strongly supporting the contribution of Cbl-b in MS pathogenesis.^122^ Several studies demonstrated that knocking out *RNF128 (GRAIL)* prevents the induction of T cell anergy in mice, setting the stage for out-of-control CD4^+^ T cells proliferation and dysregulated cytokine gene expression.^123,124^ Importantly, *Rnf128*^−/−^ mice exhibit more severe autoimmune symptoms, including enlarged spleens and mesenteric lymph nodes, massive infiltration of inflammatory cells in multiple organs, and enhanced susceptibility and severity to EAE, than WT control.^123,124^ Their findings revealed the underlying mechanism by which RNF128 hinders the progression of EAE via maintaining T cell anergy and tolerance.

#### Type I diabetes (T1D)

T1D is a chronic autoimmune disease caused by the immune-mediated destruction of pancreatic β cells, which results in insulin deficiency and hyperglycemia.^125,126^ The *RNF56* has been identified as a major susceptibility gene in the development of diabetes and other autoimmune features of the Komeda diabetes-prone rat, an animal model of T1D.^127,128^ Further study showed a significant association between one SNP in exon 12 of the *Cbl-b* gene and T1D in a large Danish T1D family material of 480 families.^129^ Subsequent findings revealed that Cbl-b deficiency precipitates T1D in most 3A9 TCR:insHEL double transgenic mice to abrogate Cbl-b-dependent T cell anergy.^130^

#### Others

Auto-inflammatory diseases are immune system hyperactivation without high-titer autoantibodies or antigen-specific T cells.^131^ In a patient with multiorgan autoinflammation, combined immunodeficiency, subclinical amylopectinosis, and systemic lymphangiectasia, the homozygous mutation in *HOIP* (HOIL-1-interacting protein, also named *RNF31*) gene at Leu 72 Pro (L72P) has been identified.^132^ That mutation is at least severely hypomorphic for destabilization of HOIP itself and the whole LUBAC complex, consisting of composed of HOIP (RNF31), Shank-associated RH domain–interacting protein (SHARPIN), and HOIL-1 (haem-oxidized IRP2 ubiquitin ligase-1, also named RNF54). In addition, the linear ubiquitination and NF-κB activation are impaired in fibroblasts derived from the patient, which can be restored by the WT *HOIP* allele for enhanced stability of LUBAC. In contrast, the patient’s monocytes are hyperresponsiveness to IL-1β from HOIP- or HOIL-1–deficient patients, which may be responsible for autoinflammation. Since the absence of one subunit of LUBAC impairs the expression of the other two subunits, the cellular and clinical phenotypes of *HOIP*-deficient patients are consistent with *HOIL-1*-deficient patients, carried biallelic loss-of-expression and loss-of-function mutations in *HOIL-1.*^133^

### Neurodegenerative diseases

Neurodegenerative disorders, including Alzheimer’s disease (AD), Parkinson’s disease (PD), Huntington’s disease (HD), amyotrophic lateral sclerosis (ALS), frontotemporal dementia (FTD), and related tauopathies, represent a major socio-economic challenge for their high prevalence yet poor treatment.^134^ Neurodegenerative diseases are characterized by the accumulation of aberrantly processed and misfolded proteins, which can be efficiently removed by quality control systems composed of the Ub-proteasome system (UPS) chaperone-mediated autophagy (CMA), and macroautophagy.^135^ Accumulating evidence has demonstrated that the abnormal RNF proteins-mediated ubiquitination, caused by genetic alterations, is involved in the pathogenesis of neurodegenerative diseases (Table 1).

#### Parkin RBR E3 ubiquitin ligase in PD and AD

PD is characterized by a relatively selective loss of dopaminergic neurons in the substantia nigra and the presence of midbrain Lewy bodies, which are protein aggregates consisting of many proteins, including α-synuclein.^136^ *Parkin*, encoding Parkin, one of the RBR E3s, is the first gene to be identified with mutations linked to autosomal recessive Parkinsonism.^137^ More than 100 mutations have been identified in Parkin, including missense point mutations, truncation mutations, large chromosomal deletions and duplications spanning one or more exons, and promoter mutations.^138,139^ The mutations in *Parkin* now represent the major cause of hereditary Parkinsonism, accounting for as much as 50% of familial early-onset PD cases and about 2–6% of late-onset PD cases.^140–142^ Genome-wide association studies have indicated 26 PD risk loci among the different *PARK* genes, including *Parkin.*^143^ A subset of the mutations in *Parkin* causes a loss of Parkin E3-ligase activity, which possibly mediates the degradation of α-synuclein and is associated with abrogation of the neuroprotective effects of Parkin.^30,144–146^ However, it is still unclear whether and how Parkin regulates the stability of α-synuclein and the role of this mechanism in PD pathogenesis.

PTEN-induced kinase 1 (PINK1) and Parkin RBR E3 ubiquitin-protein ligase play a key role in the mitophagy for directing damaged mitochondria to degradation.^147–149^ Upon detecting the reduction of mitochondrial ΔΨm caused by damage/dysfunction, the PINK1 accumulates at the outer mitochondrial membrane. Subsequently, the PINK1 recruits and activates Parkin, which mediates the ubiquitination of mitochondrial proteins, resulting in the engulfment of mitochondria into the autophagosome for degradation.^150,151^ The defective mitophagy machinery and PINK1/Parkin axis are present in human AD/PD samples and related experimental models.^152–160^ Cytosolic Parkin exhibits depletion during disease progression in AD patients, resulting in the aberrant accumulation of dysfunctional mitochondria for inadequate mitophagy capacity.^152^ The further report also showed the dysregulated protein level of Parkin in sporadic AD fibroblasts and brain biopsies.^161^ Upon mitochondria depolarization, the recruitment of Parkin to mitochondria is dramatically reduced in sporadic AD fibroblasts, implicating a defective mitophagy for insufficient labeling of damaged mitochondria. More strikingly, *Parkin* overexpression compensates for mitophagy deficiency to improve mitochondrial recycling via promoting the targeting of Parkin to the mitochondria in sporadic AD fibroblasts.

Nitrosative stress is a key pathological hallmark in PD, and S-nitrosylation of Parkin by nitric oxide contributes to defective mitophagy and consequent accumulation of damaged mitochondria in PD pathogenesis.^162–164^ The S-nitrosylation of Parkin initially activates its E3 ligase activity, leading to its autoubiquitination, which subsequently inhibits the activity of Parkin, impairing the ubiquitination of Parkin substrates, such as mitochondria proteins.^162^ Besides, the S-nitrosylation of Parkin impairs its E3 activity, and the protective effect that *Parkin* overexpression rescues cell death induced by the coexpression of α-synuclein and synphilin-1 in the presence of the proteasome inhibitor, consequently contributing to the degenerative process in PD by dysregulated the ubiquitination of many Parkin putative substrates.^164^ Rizza *et al*. further revealed that excessive S-nitrosylation of Parkin could dampen its capability to enhance mitophagy, which may be the potential mechanism involved in PD pathogenesis.^163^

Despite many challenges, Parkin, an RBR E3, offers multiple promising therapeutic targets for treating PD and relevant neurodegenerative diseases. Several pre-clinical studies show that *Parkin* gene augmentation, provided before the injury, improves disease features via viral-mediated delivery of *Parkin* in PD models.^165,166^ Lentiviral-mediated gene therapy delivery of *Parkin* into substantia nigra strikingly inhibits the α-synuclein-induced neuropathology, including preserving tyrosine hydroxylase-positive cell bodies in the substantia nigra and sparing tyrosine hydroxylase-positive nerve terminals in the striatum, in a rat model of PD.^166^ The viral overexpression of *Parkin* by adeno-associated virus (AAV) -mediated delivery into the substantia nigra also inhibits dopamine neuron death in 1-methyl-4- phenyl-1,2,3,6-tetrahydropyridine (MPTP) -intoxicated mice, a model for PD.^165^ It is worth mentioning that *Parkin* transgenic mice exhibit less reduction of neurons in the substantia nigra, induced by MPTP, especially in the old transgenic group. They further revealed that the overexpression of *Parkin* could ameliorate the MPTP-induced mitochondrial impairment in the substantia nigra of *Parkin* transgenic mice, reducing striatal α-synuclein protein in old *Parkin* transgenic mice, thereby protecting dopaminergic neurons against neurodegeneration induced by MPTP.^167^ The finding provided complicated cellular and molecular mechanisms involved in the neuroprotection of Parkin in the mice model of PD.

Enhancing Parkin-mediated mitophagy is another promising therapeutic approach to improve the consequences of mitochondrial dysfunction leading to PD pathology. A cell-based high-throughput screening identified that both T0466 and T0467 activate Parkin mitochondrial translocation in a PINK1-dependent manner to suppress mitochondrial aggregation in dopaminergic neurons differentiated from iPS cells.^168^ However, additional investigation needs to determine these compounds’ molecular targets and address their potential effects in Parkin-associated PD mammalian models. Recently identified several compounds, enhancing Parkin translocation to mitochondria without reducing the viability of cells via consistently targeting and inhibiting Rho-associated protein kinase (ROCK), activate PTEN to suppress Parkin-mediated mitophagy.^169,170^ They further indicated that the compound SR3677, the most efficacious and more selective inhibitor of ROCK2 that is enriched in brain tissue, exhibits neuroprotective effects in a *Drosophila* PD model in a Parkin-dependent manner.^169^ Their findings provided potential therapeutics for treating PD and other neurodegenerative diseases characterized by mitochondrial dysfunction. AUTEN-99 (2-(4-Phenylphenyl)-5,6-Dihydroimidazo[2,1-B][1,3]Thiazole) activates the autophagy to alleviate neurodegenerative symptoms in the *Drosophila* model of PD and also HD, represented by overexpression human mutant Parkin (R275W) and HTT protein in *Drosophila* strain, respectively.^171^ The AUTEN-99 may be a potent drug candidate for preventing and treating PD caused by certain *Parkin* mutations.

#### Others

AD is the most common type of dementia, with memory loss and cognitive dysfunction as its main symptoms, characterized by the aggregation of amyloid-β peptide (Aβ).^154,172^ The RNF146, a novel PARsylation-directed ring finger E3 ubiquitin ligase, interacts with Poly(ADP-ribose) (PAR) to inhibit the initiation of Parthanatos, a special form of cell death dependent on PAR in many human diseases, thereby exerting neuroprotective property against glutamate NMDA receptor-mediated excitotoxicity.^173,174^ In addition, *RNF146* is selectively up-regulated in highly vulnerable brain tissues of AD patients, implicating the potential function of RNF146 in the early progression of neurodegenerative diseases.^175^ Additional studies are required to determine whether the early enhanced RNF146 expression contributes to neurodegeneration of AD or protects neurons from injury in early AD. The null mutation of *Mgrn1* causes progressive spongiform degeneration in mice, similar to prion-induced neuropathology but without accumulation of protease-resistant prion protein.^176^ The further report revealed that RNF156 (MGRN1) interacts with TSG101, a key component of the endosomal sorting complex required for transport (ESCRT)-I complex, and ubiquitinates the TSG101 to facilitate cargo transport from endosome to lysosome trafficking.^177^ Since the aberrant endosomal trafficking is implicated in several neurodegenerative diseases, including progressive spongiform degeneration, they suggest that the dysfunction of endosomal ubiquitin signaling may be a pathogenic mechanism underlying spongiform neurodegeneration in *Mgrn1* null mice. The RNF182 is selectively upregulated in postmortem brains of AD patients, and the RNF182 overexpression triggers cell death of neurons.^178^ RNF182 physically interacts with ATPase 16 kDa proteolipid subunit (ATP6V0C), a key component of gap junctions and neurotransmitter release channels, and ubiquitinates ATP6V0C for proteasomal degradation. They further hypothesized that the upregulation of RNF182 in AD patients’ brains might contribute to AD’s pathophysiology by mediating the ubiquitination and degradation of ATP6V0C.

A whole-exome and targeted sequencing performed in a patient consanguineous family with a syndrome of cerebellar ataxia, dementia, and hypogonadotropic hypogonadism, showed that the loss-of-function mutations in *RNF216* may cause that disease.^179^ Their in-vivo study further revealed that the knockdown of *Rnf216* in zebrafish embryos results in eye, optic tectum, and cerebellum defects. The virtually complete loss of neuronal loss is observed in hippocampal regions CA3 and CA4 in patients with *RNF216* mutations. Whole-exome sequencing in seven nuclear families characterized by progressive ataxia and deafness (AR-LAD) combined with fibrotic cardiomyopathy and hepatopathy as major associated features recently revealed that 2 homozygous missense variants (p.R363Q and p.R365Q) in RNF220 may be the causative event underlying this novel syndromic leukodystrophy AR-LAD, a neurodegenerative diseases.^180^ The p.R363Q and p.R365Q mutations affect RNF220 subcellular localization and enhance cytoplasmic aggregation. Besides, RNAi-mediated downregulation of *Drosophila RNF220* also significantly and specifically affects the proper localization of lamin B1 fly orthologue, consequently facilitating lamin B1 aggregation and neurodegeneration. In-vitro data showed that the RNF220 physically associates with lamin B1, while AR-LAD mutations, particularly p.R365Q, dramatically weaken that interaction. Whether the dysfunction of lamin B1 by RNF220 carrying p.R363Q and p.R365Q mutations may be an underlying pathogenic mechanism of AR-LAD needs further investigation.

## RNF family proteins in viral infection

Viruses pose a major threat to global health, particularly the SARS-CoV-2 emerging in recent years. Host antiviral innate and adaptive immune responses provide a powerful defense against invading viruses. Strict and precise immune system regulation is fundamental for effective pathogen clearance and preventing overreacting host damage.^181^ The innate immune system recognizes viral pathogen-associated molecular patterns (PAMPs) depending on germline-encoded receptors known as pattern recognition receptors (PRRs), including Toll-like receptors (TLRs), retinoic-acid inducible gene-I (RIG-I)-like receptors (RLRs), C type lectin receptors (CLRs), as well as several inflammasomes and DNA sensors, and then produce IFN and proinflammatory cytokines by a series of signaling pathways to limit the viral spread and regulate the action of the ensuing adaptive immune response.^182^ Type I and III IFNs are powerful antiviral agents. They efficiently induce hundreds of interferon-stimulated genes (ISGs) production through the JAK-STAT signaling pathway to establish an antiviral state by controlling and restricting viral infection and replication.^183^ RNF proteins act as critical regulators of PRRs signaling pathways, such as TLRs, RLRs, the DNA sensor cyclic GMP-AMP synthase (cGAS), and the downstream JAK-STAT signaling pathway, as well as acquired immunity. We will discuss how other RNF proteins regulate these four major innate signaling pathways and adaptive immune responses during viral infection diseases.

### TLR signaling pathway

**Fig. 2:**
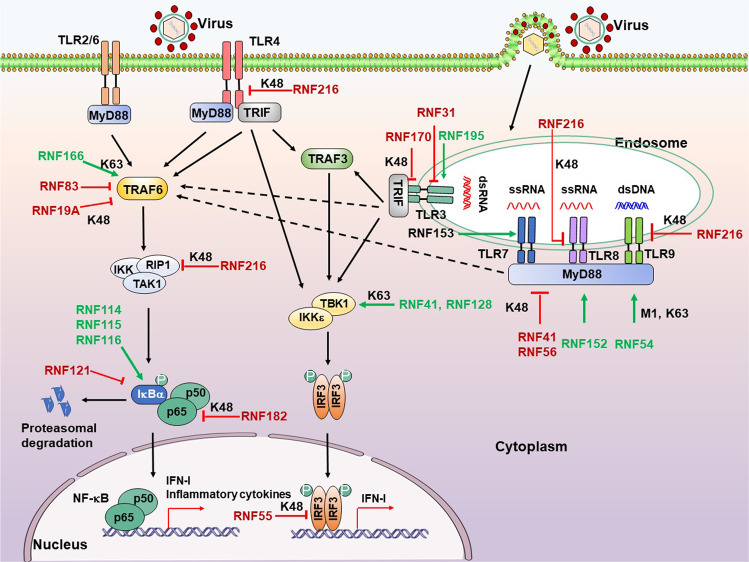
RNF proteins regulate the TLR signaling pathway. TLRs locate at both plasma membrane and endosomes. TLRs sense different ligands like viral nucleic acids and viral envelope glycoproteins and recruit TRIF and MyD88 to transduce signals, ultimately activating IRFs and NF-κB to product IFN-I and proinflammatory cytokines. RNF proteins positively (green arrows) or negatively (red T-shaped solid line) regulate multiple steps downstream of the TLR signaling pathway. P, phosphate

TLRs are the first known PRRs, the core aspects of innate immune responses. TLRs are transmembrane proteins suitable for detecting viral components outside of cells and in cytoplasmic vacuoles after phagocytosis or endocytosis to induce type I IFN (IFN-I) and proinflammatory cytokines to resist virus invasion.^184^ The different subcellular locations of TLRs allow the host to detect infection throughout the viral life cycle.^185^ TLR3, TLR7, TLR8, and TLR9 are located on the endosomal membrane and detect viral nucleic acids. TLR1/2 and TLR4, presenting on the cell membrane, detect viral envelope proteins. All TLRs, except TLR3, recruit the adaptor molecule Myeloid differentiation factor 88 (MyD88) upon activation, and the latter subsequently engages E3 ubiquitin ligase TNF receptor-associated factor 6 (TRAF6). The activated TRAF6, through autoubiquitination, interacts with the IKKα/β/γ, TAK1, and RIPI complex, ultimately resulting in the activation of NF-κB to product IFN-I and proinflammatory cytokines.^184^ However, TLR3 (and TLR4) utilizes the adaptor molecule TIR-domain-containing adaptor-inducing IFN-β (TRIF) to activate IRF3 via TBK1/IKKε-mediated phosphorylation. Phosphorylated IRF3 dimer then enters the nucleus to initiate the IFN-I expression.^186^ Interestingly, RNF proteins regulate several steps in TLR signaling pathways (Fig. 2).

#### TLRs

The deficiency of *Rnf170* significantly enhances the induction of IFN-I in peritoneal macrophages in response to EMCV infection, reduces the EMCV titers and viral RNA replicates in the heart and brain, attenuates inflammatory cell infiltration and tissue damage in heart tissue, as well as improves mice survival.^187^ In poly(I:C)-stimulated murine cells, RNF170 can bind the TLR3 and exert its E3 ligase function, which mediates the K48-linked poly-Ub of K766 in the TIR domain of TLR3 and promotes the proteasomal degradation of TLR3. This study first identified the substrates and inhibitory function of RNF170 in TLR3-mediated innate immune responses, thereby enhancing viral infection and pathogenesis and providing a potential target for controlling TLR3-related inflammatory diseases. Interestingly, according to the Gene Expression Omnibus database (accession no. GSE5220 and GSE3194), the RNF170 expression is significantly upregulated in monocytes during HIV infection while increased first and then decreased in fibroblasts during cytomegalovirus infection.

Similarly, RNF31 (HOIP), one component of the LUBAC, negatively modulates TLR3 signaling induced by poly(I:C) and subsequent expression of IFNs and proinflammatory cytokines through interacting with the dsRNA-induced TLR3- signaling complex (TLR3-SC).^188^ The RNF31 is also required for limited TLR3-mediated cell death induced by poly(I:C) to prevent the development of immunodeficiency and autoinflammation. This study identifies a molecular mechanism of how RNF31 limits TLR3-induced cell death. Of note, HOIP-mutated patients present with recurrent bacterial infections and hyper-inflammation,^132^ further supporting the potential therapeutic targets of RNF31 for suppressing TLR3-related inflammatory diseases.

In contrast, Chang *et al*. have demonstrated that the deficiency of RNF195 impaired TLR3-mediated IFN response in a cell-type and stimulus-dependent manner. The *Mex3b*^-/-^ mice dramatically decrease serum IFN-I after poly(I:C) administration and enhance the resistance to poly(I:C)-induced death. Upon poly(I:C) stimulation, the RNA-binding protein RNF195 (Mex3B) serves as a coreceptor of TLR3 by interacting with viral dsRNA to increase the dsRNA-binding activity of TLR3.^189^ Besides, RNF195 also enhances TLR3 stability via promoting proteolytic processing of TLR3, which is required for later activation. That finding suggested a potential mechanism for presenting endosomal dsRNA to TLR3. This study also provided a potential target for controlling TLR3-related inflammatory diseases.

RNF216 (Triad3A/ZIN) interacts with the cytoplasmic tail (CT) of several TLRs and promotes proteolytic of TLR4 and TLR9 through K48-linked poly-ubiquitination to limit signaling mediated by respective TLRs but did not affect TLR2 expression or signaling.^190^ They identified that RNF216 acts as an E3 ubiquitin-protein ligase, and additional studies are needed to address whether RNF216 incorporates into the TLR4/9 signalosomes. Furthermore, RNF216 is also a negative regulator of TLR8 signaling by targeting TLR8 for K48-linked ubiquitination and degradation after stimulation by RNA ligands.^191^ The RNF216 expression significantly decreases in Critically Ill Subjects at Risk for or with acute respiratory distress syndrome (ARDS), implicating that the downregulation of RNF216 may contribute to potentiate excessive inflammation in subjects with or at risk for ARDS. Their findings provided potential targets at the TLR8–RNF216 signaling axis for the prospective therapeutic strategy for severe lung injury. RNF153 (MARCH5) particularly binds TANK, a negative regulator of TLR7 signaling, and catalyzes the K63-linked poly-Ub TANK, which modification prevents TANK from inhibiting TRAF6, thereby potentiating TLR7-mediated NF-κB activation.^192^ The study identified a novel function of mitochondrial protein MARCH5, an essential and positive modulator of TLR7 signaling.

#### MyD88

MyD88, a vital and canonical adaptor protein downstream of the TLRs signaling pathway, comprises a C-terminal Toll-interleukin-1 receptor (TIR) domain, mediating the interaction with receptors or adaptors, an N-terminal death domain required for association with the IRAK family.^193,194^ RNF proteins modulate the stability of MyD88 through ubiquitination and proteasome-dependent degradation, as well as modify the oligomerization of MyD88, thereby influencing the downstream pathways. Nrdp1-transgenic mice resist LPS-induced pro-inflammatory cytokines production in vivo, thereby protecting mice from lethal LPS-induced liver injury. The RNF41 (Nrdp1) directly interacts with MyD88, facilitating its K48-linked poly-Ub and final degradation to negatively regulate MyD88-mediated activation of NF-κB and AP1, suppressing the production of proinflammatory cytokines.^195^ This report suggested that the RNF41 is one of the promising new therapeutic targets for controlling inflammatory diseases. RNF56 (Cbl-b) directly binds MyD88, leading to degradation of MyD88 via poly-ubiquitination and the subsequent inhibition of MyD88-mediated inflammatory responses.^196^ A previous study has indicated that the deficiency of Cbl-b potentiates the sepsis-induced acute lung inflammation and mortality by enhancing the MyD88-dependent acute inflammatory response to sepsis.^197^ The RNF56 may be a new potential target for regulating the acute inflammatory response to sepsis. However, RNF152 associates with the adaptor protein MyD88 and enhances the oligomerization of MyD88, which positively regulates the TLR/IL-1R signaling pathway.^198^ Besides, *Rnf152*^*−/−*^ mice present attenuated inflammatory cytokine production, delayed onset of death, and low percentage of lethality, supporting the potential therapeutic target of RNF152 for suppressing MyD88-dependent inflammatory diseases. Additional studies are required to determine how the membrane localization of RNF152 affects its function on MyD88-mediated signaling. Interestingly, RNF54 (HOIL-1), another component of the LUBAC, catalyzes the monoubiquitylation of MyD88, which modification initiates the de novo synthesis of K63- and M1-based poly-Ub chains of MyD88, leading to activation and oligomerization of MyD88.^199^ This striking study revealed that RNF54 catalyzes the formation of oxyester bonds between Ub and targeted protein and firstly described the 9^th^ type of ubiquitin linkage mediated by RNF54.

#### TRAF6

TRAF6 is an adaptor protein belonging to the TRAF family, which can activate TLRs mediated downstream signaling pathways to ultimately causes the activation of NF-κB and subsequent expression of IFN and proinflammatory cytokines. TRAF6 interacts with TAK1 and IKK complex. The latter then phosphorylates the IκBα, causing the ubiquitination and degradation of IκBα to release NF-κB.^200^ Evidence showed that RNF166 interacts with TRAF6, enhancing the K63-based ubiquitination of TRAF6 to potentiate RNA virus-induced IFN-β, leading to inhibition of RNA virus infections.^201^ However, Takeshita *et al*. identified that RNF83 (TRAF4) physically associates with TRAF6 to disrupt the function of TRAF6, negatively regulating TRAF6-mediated NF-κB and IFN-I responses.^202^ Interestingly, activation of TLR appeared to induce TRAF4 expression, which in turn suppressed TRAF6-dependent TLR signaling in a negative-feedback manner. Their study characterizes the novel function of TRAF4 that may serve to antagonize TRAF6-mediated cellular signaling. Strikingly, NLRP11 functions as an adaptor to recruit RNF19A, which subsequently promotes the K48-linked ubiquitination and degradation of TRAF6, leading to attenuation of inflammatory responses.^203^ Wang’s study revealed a novel mechanism by which NF-κB-induced NLRP11 suppresses TRAF6-mediated signaling pathways via recruiting RNF19A, preventing dysregulated inflammatory response through this negative feedback loop.

### RLR signaling pathway

**Fig. 3:**
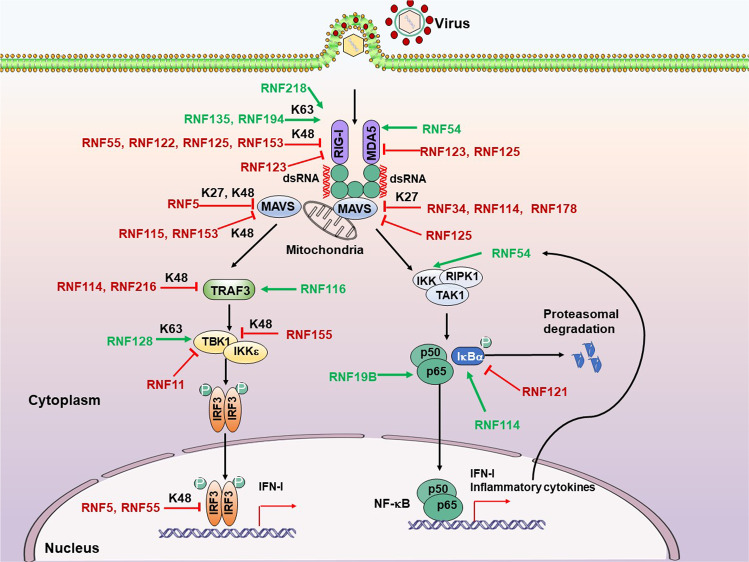
RNF proteins modulate the RLR signaling pathway. Following the recognition of distinct dsRNA features by RIG-I and MDA5, their CARDs recruit and activate the downstream adaptor protein MAVS to trigger IRF3 and NF-κB activation, resulting in the production of IFN-I and inflammatory cytokines. RNF proteins emerged as key regulators via positively (green arrows) or negatively (red T-shaped solid line) modulating the RLR signaling pathway. P, phosphate

The RLRs encompass three members: RIG-I, melanoma-differentiation-associated gene 5 (MDA5), and laboratory of genetics and physiology 2 (LGP2).^204^ RLRs are highly homologous in structure. DeXD/H-box RNA helicases and a zinc-binding domain at the C terminal of the RLR family are essential sensors of RNA virus infection.^204^ Following the recognition of distinct dsRNA features, the N- terminal caspase activation and recruitment domains (CARDs) of RIG-I and MDA5 but not LGP2 can recruit and activate the downstream adaptor mitochondrial antiviral signaling (MAVS) protein, located at the outer mitochondrial membrane.^205,206^ Subsequently, the activated MAVS protein recruits downstream proteins TBK1 and IKK-related kinases IKKε to phosphorylate and activate IRF3/7 via the TRAF3–TANK–TBK1 axis, as well as canonical IKKα/β/γ complex to promote degradation of the NF-κB inhibitor IκBα through the FADD–RIP1–IKK axis, leading to NF-κB activation, which ultimately accompanies some other transcription factor to stimulate the expression of IFN-I and proinflammatory cytokines.^207–209^ Several studies have identified that RNF proteins emerged as key regulators of RLR-triggered antiviral response (Fig. 3).

#### RIG-I and MDA5

RLRs are best represented by RIG-I and MDA5, which have been proven to play a crucial role during RNA viral infection and activate a series of antiviral signaling pathways, leading to the production of IFNs and proinflammatory cytokines.^210^ RNF proteins are critical in regulating the RIG-I/MDA5 and downstream signaling pathways. Several RNF proteins negatively regulate the RIG-I/MDA5 stability by attaching the classical degradative K48-linked poly-ubiquitination to targeted RIG-I/MDA5. RNF55 (c-Cbl), recruited with RIG-I to Siglec-G that is upregulated following RNA viruses infection, conjugates K48-linked poly-Ub to K813 residue of RIG-I to promote RIG-I degradation in a proteasome-dependent manner.^211^ This study provided a novel negative feedback regulation of RIG-I-mediated antiviral innate immunity through PTM of RIG-I. Previous studies have indicated that Kaposi’s sarcoma-associated herpesvirus (KSHV) could hijack RNF55 to promote viral internalization during infection of endothelial cells.^212^ However, whether the KSHV or other viruses hijack the RNF55-mediated inhibition of RIG-I to antagonize its antiviral function needs to be further addressed. During the early phase of RNA virus [vesicular stomatitis virus (VSV) or Sendai virus (SeV)] infection, the mRNA and protein levels of RNF122 significantly increase and then decrease at late stage.^213^ RNF122 directly binds to RIG-I and transfers K48-linked ubiquitination to Lys115 and Lys146 residues of RIG-I CARDs to promote RIG-I degradation via proteasomes inhibiting the IFN-I response against RNA viruses infection.

Besides, Arimoto et al. have noted that RNF125 mediates the K48-linked ubiquitination and subsequent degradation of RIG-I via proteasomes and directly interacts with MDA5 and MAVS to suppress MDA5- as well as MAVS- mediated signaling in a Ub conjugation-dependent manner, thereby dramatically repressing IFN-I production to potentiate viral infection.^214^ In addition, after IFN-I or poly(I:C) treatment, the RNF125 production is upregulated, inhibiting RIG-I-mediated IFN-I responses in a negative-feedback manner. The human bocavirus (HboV) VP2 physically associates with RNF125 to block RNF125-mediated RIG-I degradation, promoting the IFN-I production, which may enhance HboV persistent infection.^215^ RNF153 (MITOL or MARCH5), the only member of the MARCH family localized in mitochondria, attaches the K48-linked poly-Ub chain on RIG-I for proteasomal degradation.^216^ The SPLA/Ryanodine receptor (SPRY) and coiled‐coil domain of RNF123 interact with the N-terminal CARD domains of RIG-I/MDA5 and compete with the downstream adaptor MAVS for RIG-I/MDA5 CARD binding to significantly suppress RLR‐mediated antiviral signaling, thus enhancing the replication of several RNA viruses.^217^ That study highlighted a novel mechanism by which RNF123 inhibits RIG-I/ MDA5- mediated innate antiviral signaling in a catalysis‐independent manner.

Conversely, several RNF proteins are also responsible for potentiating the activation of RIG-I/ MDA5 and their downstream signaling via attachment of K63-linked ubiquitination. RNF135 (Riplet/ REUL) directly interacts with RIG-I to catalyze the K63-linked ubiquitination of the C-terminal region of RIG-I, which modification differs from the N-terminal ubiquitination of RIG-I by TRIM25, thereby facilitating RIG-I activation.^218–220^ Their knockout study revealed that RNF135 is essential for RIG-I activation and antiviral IFN responses to RNA virus infection in vivo.^221^ The *Riplet*^−/−^ mice were more susceptible to VSV infection than wild type for attenuated RIG-I-dependent IFN-I production at the early stage of VSV infection. They further identified that RNF135 functions upstream of TRIM25 for RIG-I ubiquitination and activation,^222^ in which RNF135-mediated K63-linked polyubiquitination of the RIG-I repressor domain to release its 2 N-terminal CARDs, which is then modified by TRIM25 to activate RIG-I. Of note, Hepatitis C virus (HCV) protease NS3-4A targets RNF135 and reduces the protein level of RNF135, suppressing RNF135-mediated release of RIG-I RD autorepression, thereby abolishing the RIG-I activation and antiviral IFN production. Interestingly, it has been reported that the Influenza A virus (IAV) NS1 protein targets RNF135-dependent ubiquitination and activation of RIG-I for suppressing antiviral IFN production.^223^ These immune escape strategies implicate the biological importance of RNF135-mediated RIG-I activation during viral infection, which provides potential therapeutic targets of RNF135 for controlling HCV/IAV infection and HCV-related diseases.

Although accumulating studies in cells showed that TRIM25 knockout/knockdown is essential for RIG-I activation and significantly impairs cytokine responses to diverse stimuli,^219,224–229^ several more recent studies have challenged this model and demonstrated that upon virus infection, the RNF135 but not TRIM25 is responsible for RIG-I activation.^230–232^ Cadena et al. proposed a novel mechanism that RNF135 activates RIG-I in both ubiquitin-dependent and filament bridging-dependent manners, which work to discriminate the length of dsRNA recognized by RIG-I.^232^ Mechanistically, RNF135 binds the core RIG-I filaments, comprising RIG-I and short dsRNA end (>20 bp), and catalyzes ubiquitination of multiple sites on the RIG-I, including 2 CARDs. On longer dsRNAs (~40–500 bp), RNF135 can also cross-bridge filamentous oligomers of RIG-I to induce higher-order receptor “clustering”, amplifying RIG-I-mediated antiviral signaling by enhancing the local concentrations of RIG-I, tetramerization of RIG-I 2 CARDs, and nucleation of MAVS. They provide a previously unrecognized mechanism by which nucleic acid sensor adopts E3 ligase to discriminate the length of foreign nucleic acid. Additional studies are required to determine how RNF135 identifies RIG-I filaments but not MDA5. Importantly, Oshiumi and colleagues recently indicated that RNF135 is a general factor for RIG-I activation and RIG-I-dependent cytokine expression, while TRIM25 acts as a regulator/activator of RIG-I activation in a cell-type dependent manner.^233^ Their finding would resolve the previous apparent contradiction of whether the TRIM25 is essential for RIG-I activation. They also revealed that the RNF135 mediated K63-linked poly-ubiquitination of LGP2 reduces RIG-I-dependent IFN-I at a late phase of viral infection to avoid excessive cytokine expression. During SARS-CoV-2 infection, RNF135 knockout decreases the IFN-I production and dramatically enhances the viral replication. These findings suggested that the RNF135 may be a new potential target for controlling the pandemic coronavirus disease 2019 (COVID-19).

*Mex3c*^−/−^ mice display growth retardation after birth, and the IFN production, induced by the RNA virus, is severely impaired, resulting in elevation of viral replication in the *Mex3c*-deficient peritoneal macrophages.^234^ Besides, RNF194 (MEX3C), an RNA binding protein, preferentially colocalizes with viral RNA and RIG-I, which consequent proximity allows RNF194 to transfer the K63-linked poly-ubiquitination to N-terminal CARDs of RIG-I to facilitate RIG-I–mediated antiviral signaling. Further studies are required to address the role of RNF194 in discriminating self-RNAs and viral RNAs. RNF218 (MUL1/MAPL), localized to the mitochondria, SUMOylates the RIG-I in an energy and temperature-dependent manner to activate RIG-I, which modification of RIG-I exposes the 2 CARDs to promote the assembly of the MAVS signaling complex.^235^ Mouse embryonic fibroblasts lacking MAPL significantly reduce IFNβ production, thereby potentiating the SeV replication. Liao and colleagues reported that the csRNF114, RNF114 from Chinese sturgeon (*Acipenser sinensis*), can be induced with poly(I:C) stimulation and positively regulates the RLR signaling.^236^ RNF54 (HOIL-1) is suppressed for IFN induction via the MDA5-MAVS-TBK1-IRF3 axis after Murine norovirus (MnoV) infection, while the relevant targets of RNF54 in the MDA5 signaling pathway remain unknown.^237^ The in vivo data also showed that RNF54 is required for IFN responses to restrict acute replication and enhance systemic clearance of MnoV. Their findings provided potential targets at the MDA5-RNF54 signaling axis for a prospective therapeutic strategy for acute gastroenteritis caused by human norovirus.

#### MAVS

MAVS (also known as IPS-1, VISA, and Cardif) contains a C-terminal transmembrane domain and an N-terminal CARD domain interacting with RIG-I and MDA5 CARD domain to transduce antiviral signaling, which homotypic interaction activates MAVS to form prion-like aggregates.^208^ The activated MAVS aggregates work as a scaffold to recruit several TRAFs, leading to IRF3/7 activation for IFNs induction through the TRAF3-TBK1 axis and NF-κB activation for proinflammatory cytokines production via RIPK1-IKK-TAK1 axis.^238^ It has been demonstrated that several RNF proteins are responsible for suppressing MAVS activation and the downstream signaling pathways by negatively modulating the MAVS stability. RNF5, located at mitochondria, interacts with MAVS to attach K48-linked poly-Ub at Lys362 and Lys461 of MAVS for its proteasomal degradation after viral infection, negatively regulating IFN-I induction.^239^ Sun et al. recently reported that the Newcastle disease virus (NDV) V protein recruits RNF5 to mediate MAVS proteasomal degradation through Lys 362 and Lys 461 ubiquitin to suppress IFN signaling.^240^ In addition, Zeng et al. revealed that the PB1, a subunit of RNA polymerase of IAV, interacts with RNF5 and MAVS to enhance the RNF5 mediated K27-linked poly-ubiquitination of MAVS, which is recognized by an autophagic receptor, neighbor BRCA1 (NBR1), leading to autophagic degradation of MAVS.^241^ Their findings identified the evasion strategies that involve the RNF5-mediated ubiquitination and proteasomal/autophagic degradation of MAVS, which benefits NDV/IAV proliferation during infection.

In addition, RNF34 physically binds to MAVS to attach K27/K29-linked ubiquitination of MAVS at Lys297, 311, 348, and 362 Arg, which is recognized by the cargo receptor NDP52 for autophagic degradation of damaged mitochondria with enriched MAVS aggregates.^242^ The RNF34‐mediated ubiquitination of MAVS generates a signal for selective mitophagy to clear damaged mitochondria upon VSV infection. Their findings elucidated that the RNF34‐mediated autophagic degradation of MAVS links to the innate immune response, mitochondrial homeostasis, and viral infection. Several studies also identified that the RNF114/LjRNF114 directly associates with MAVS and targets it for proteasomal degradation via K27-linked ubiquitination to inhibit RLR-mediated antiviral signaling.^243,244^ However, *Rnf114*^−/−^ mice failed to display enhanced resistance to RNA virus infection. Based on the above findings, further investigation is needed to determine the paradoxical roles of RNF114 in RLR signaling pathways. Jin et al. discovered that the RNF178 (MARCH 8) is recruited by tetherin to negatively regulate MAVS-mediated IFN-I responses through attaching the K27-linked poly-ubiquitination of MAVS at Lys7, which modification is recognized by NDP52 for autophagic degradation of MAVS.^245^

A recent study indicated that RNF115 (BCA2) continuously associates with and catalyzes the K48-linked ubiquitination of MAVS for proteasomal degradation of homeostatic MAVS in uninfected cells.^246^ The in vivo data showed that the deficiency of RNF115 significantly promotes IFN-I production, inhibiting the RNA viruses replication. Upon RNA viruses infection, the RNF115 expression is significantly upregulated at transcriptional, translational, and posttranslational levels, which represses MAVS-mediated IFN-I response, thereby enhancing the RNA viruses replication in a positive feedback manner. Yoo’s group revealed that RNF153 (MARCH5) interacts with activated MAVS oligomer but not monomeric MAVS to promote the K48-linked ubiquitination-mediated degradation of MAVS aggregates, decreasing MAVS-mediated IFN signaling.^247^ That study suggests that RNF153 modulates the MAVS signaling via timely degradation of activated MAVS oligomers to switch off the persistent activation of MAVS. As mentioned above, RNF153 also targets and degrades the RIG-I oligomer.^216^ That dual inhibitory function of RNF153 on innate antiviral immunity can efficiently prevent excessive immune responses and subsequent diseases, such as SLE. A higher molecular weight MAVS aggregate in some SLE patients further supports the potential diagnostic and therapeutic targets of RNF153 for controlling autoimmune diseases.

#### TRAF3

TNF receptor-associated factor 3 (TRAF3) is an adaptor protein belonging to the TRAF family activated by RIG-I and MDA5 to mediate downstream signaling pathways.^248^ Besides TRAF6, RNF166 also promotes the production of RNA virus-induced IFN-β via associating with TRAF3 and enhancing the ubiquitination of TRAF3, which modification enhances the binding of downstream molecules to TRAF3.^201^ This study revealed a critical role of RNF166 in enhancing RNA virus-triggered IFN production by potentiating the ubiquitination of both TRAF3 and TRAF6. A recent study, controversial to a previous study, revealed that the LjRNF114, RNF114 from *Lateolabrax japonicus*, interacts with TRAF3 and transfers the K48-linked ubiquitination to TRAF3 for proteasomal degradation to modulate the RLR-mediated antiviral signaling negatively.^236,243^ Interestingly, their study also indicated that Red-Spotted grouper nervous necrosis virus (RGNNV) infection enhances the expression of LjRNF114, suppressing the RLR-dependent IFN production, thus potentiating the RGNNV replication in a positive feedback manner. This report provided a potential target at LjRNF114 for a prospective therapeutic strategy against nervous necrosis virus infection, which causes more than 90% mortality in the larval stage of fish. Besides function in TLRs, RNF216 physically interacts with TRAF3 and catalyzes the K48-linked ubiquitination of TRAF3 for proteasomal degradation to negatively modulate the RLR-mediated antiviral response against RNA virus infection.^249^

### cGAS-STING signal pathway

**Fig. 4:**
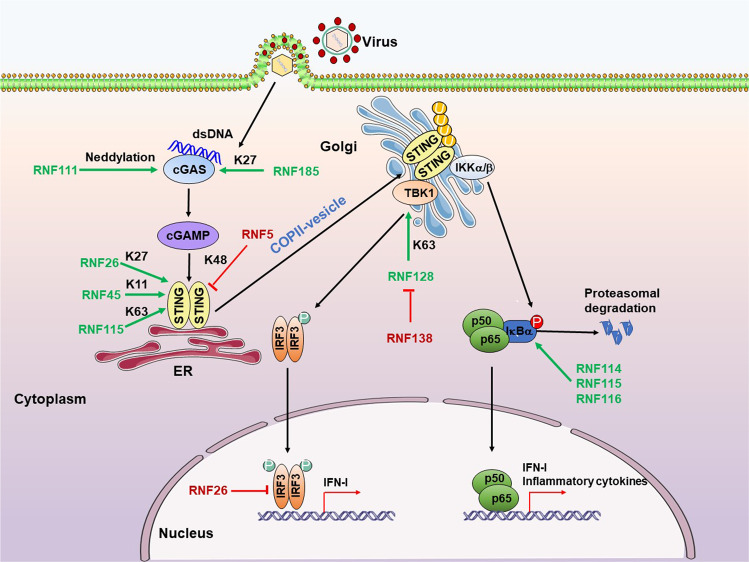
RNF proteins orchestrate the cGAS-STING signaling pathway. Upon activation by dsDNA, cytosolic cGAS triggers STING activation and translocation through synthesizing cGAMP, consequently inducing IFN-I and inflammatory cytokines production. RNF proteins can target multiple steps in the DNA sensing signal pathway. Overview of the RNF proteins that positively (green arrows) or negatively (red T-shaped solid line) cGAS-STING signaling. P, phosphate; U, ubiquitin

Various cytosolic viral DNA sensors have recently been identified, with cGAS being the most prominent.^250,251^ Upon DNA binding, cGAS synthesizes the second messenger molecule, cyclic GMP-AMP (cGAMP), which binds and activates the stimulator interferon genes (STING) via conformational change preformed STING dimer, translocating the STING from ER to Golgi through COPII-mediated vesicles. The activated STING recruits and activates the TBK1 and IKKβ to promote the nuclear importation of IRF3 and NF-κB, respectively, culminating in the production of IFN-I and proinflammatory cytokines.^252,253^ Although the characterization of PTMs that regulate the cGAS-STING pathway is still in its infancy, recent studies have demonstrated that several RNF proteins modulate cGAS-STING-dependent antiviral signaling (Fig. 4).

#### cGAS

cGAS (MB21D1/C6orf150) is identified as one of the most important cytosolic DNA sensors for detecting dsDNA in a DNA length-dependent manner.^254^ Upon binding to dsDNA, cGAS utilities ATP and GTP as substrates to catalyze the production of 2′3′‐cGAMP, then binding to STING on ER membrane to facilitate STING-mediated antiviral immune responses.^255^ RNF proteins have emerged as vital molecules in regulating cGAS-mediated antiviral immunity. A more recent study has exhibited the poly-neddylation of cGAS by the Ube2m-RNF111 axis, which modification potentiates cGAS dimerization and its DNA-binding ability to modulate the cGAS-mediated immune responses against HSV-1 infection positively.^256^ The protein level of RNF111 is upregulated while not affected by RNA level, which facilitates cGAS-dependent IFN-I response against DNA virus infections in a negative feedback manner. Their findings revealed an indispensable role of RNF111-mediated neddylation modification for cGAS activation and cGAS-mediated restriction of DNA pathogens infection. RNF185, located on ER, interacts with cGAS and catalyzes the K27-linked poly-ubiquitination of cGAS at Lys173/Lys384 residues, enhancing the cGAS enzymatic activity through promoting the recruitment of TBK1 to STING, which ultimately promotes that IFN responses against HSV-1 infection.^257^ Further investigation is required to determine the role of K27-linked polyubiquitination of cGAS in cGAS dimerization or oligomerization.

#### STING

STING (also known as TMEM173, MITA, ERIS, and MPYS) as an ER‐located adaptor protein contains four N-terminal transmembrane helices and one globular C-terminal cytosolic domain (CTD), which domain is responsible for recruiting and activating the TBK1 and IKKβ.^258–260^ Several RNF proteins positively or negatively regulate the stability or activity of STING via ubiquitination modification. RNF5 associates with STING and ubiquitinates STING at Lys150 for K48-linked ubiquitination and proteasomal degradation after viral infection,^261^ denoting the important role of RNF5 in dampening virus-triggered IFN-I induction by targeting both MAVS and STING degradation.

In contrast, Qin et al. have delineated that RNF26, mainly located at ER and constitutively interacting with STING, promotes the K11-linked polyubiquitination of STING at lys150, which modification unlocks the virus-triggered K48-linked poly-ubiquitination of STING to prevent its proteasomal degradation.^262^ This regulatory mechanism is required for rapid and efficient induction of both IFN-I and proinflammatory cytokines at an early stage of viral infection. RNF45 (AMFR, gp78), an ER-located E3 ligase, mediates K27-linked poly-ubiquitination of STING at Lys150 and in response to HSV DNA.^263^ The K27-linked polyubiquitin moieties act as an anchoring platform to recruit and activate TBK1, enhancing STING-mediated antiviral signaling. The *Amfr*^−/−^ mice are more susceptible to HSV-1 infection than wild type due to attenuated STING-dependent IFN-I production. After HSV-1 infection, RNF115 (BCA2) interacts with STING to catalyze the K63-linked ubiquitination of STING, potentiating the aggregation of STING and STING-dependent IFN-I responses.^246^ The in vivo data denoted that the deficiency of RNF115 impaired IFN-I production, potentiating the replication of DNA viruses. Their studies revealed dual roles of RNF115 in innate antiviral responses by negatively modulating homeostatic MAVS and positively regulating STING activation.

### TBK1-IRF3 axis

TBK1, a multifunctional serine/threonine kinase protein belonging to IκB Kinase(IKK) family, is primarily responsible for IFN-I response against viral or aberrant cytoplasmic nucleic acids.^264^ Upon pathway stimulation, TBK1 is recruited by adaptor proteins that deliver TBK1 to specific signaling complexes and direct subcellular localization.^265^ TBK1 activation is subject to multiple layers of regulation, including dynamic recruitment by adaptors, alterations in subcellular localization, and PTMs involved in phosphorylation, hydroxylation, acetylation, and ubiquitylation.^266^ Activated TBK1 directly phosphorylates and activates IRF3, promoting its homodimerization and translocation into the nucleus to induce the expression of antiviral IFN-I. Accumulating evidence has identified that many RNF proteins modulate the TBK1-IRF3 axis (Figs. 2–4).

The previous report has indicated that the RNF11 directly interacts with TBK1/IKKε and blocks TRAF3-mediated K63-linked poly-ubiquitination by abolishing the interaction between TRAF3 and TBK1/ IKKε for inhibition of the RIG-I/MDA5-dependent IFNβ production in a catalysis‐independent manner.^267^ Besides, the mutant Rnf11 in Belgian Blue Cattle contributed to premature lethality caused by uncontrolled inflammation in the respiratory tract, which in vivo evidence supported the potential therapeutic target of RNF11 for regulating inflammatory diseases.^268^ Their studies implicated that RNF11 might function as an adaptor or scaffold protein to suppress antiviral innate immune responses. RNF41 (Nrdp1) also directly interacts with TBK1 to catalyze K63-linked poly-Ub for promoting TRIF-dependent activation of TBK1 and IRF3, thereby enhancing the TLR-mediated IFN-I production.^195^ Further investigations showed that the RNF41-mediated promotion of IFN-I responses could prevent VSV infection of macrophages, and in vivo evidence also showed that the serum IFN-β concentrations in DN-Nrdp1-transgenic mice (overexpression of an Nrdp1 mutant lacking its E3 ubiquitin ligase activity) is lower than Nrdp1-transgenic or wild-type mice, leading to higher viral loads in livers. Hence, their finding identified the dual roles of RNF41 in regulating TLR-stimulated signaling by facilitating the degradation of MyD88 and the activation of TBK1, which eventually attenuates inflammatory cytokine production and enhances IFN-I production, respectively.

Besides, RNF128 is also directly associated with TBK1 to catalyze its K63-linked poly-ubiquitination, positively regulating the TBK1 activation, IRF3 activation, and IFN-β production after both RNA-virus and DNA-virus infection.^269^ The serum protein abundance of IFN-β in *Rnf128*^−/−^ mice is lower than wild type after VSV or HSV-1 infection, while the viral loads and replications are dramatically higher in *Rnf128*^−/−^ mice, and thus severe injuries are observed in the lungs of *Rnf128*^−/−^ mice that infected with VSV. In addition, both mRNA and protein levels of RNF128 are upregulated after RNA or DNA viruses infection, while this upregulation could be repressed by blocking IFNβ signaling with an antibody to IFNRA1, which suggests that RNF128 is one of the ISGs. Their findings provided a potential target at RNF128 for controlling SLE and other autoimmune diseases. Furthermore, a recent study reported that the ASFV Pi215L, a viral E2 enzyme, potentiates the interaction between RNF128 and RNF138 to promote the RNF128 degradation in a catalysis‐independent manner, thereby attenuating the RNF128-mediated K63-linked poly-ubiquitination of TBK1 to inhibit cGAS-mediated IFN-I responses against to DNA virus infection.^270^ The findings identified a novel strategy adopted by ASFV to evade host antiviral innate immunity via hijacking the RNF proteins-mediated ubiquitination process. The TRAF3 Interacting Protein 3 (TRAF3IP3), associated with TRAF3 and TBK1, is recently identified to recruit RNF155 (DTX4) to TBK1 to promote TBK1 degradation via K48-linked poly-ubiquitination, leading to the reduction of RLR-mediated IFN-I production.^271^

IRF3, located in the cytoplasm in an inactive form, appears to be constitutively expressed in cells, which is required for rapid and efficient antiviral innate responses during an early stage of viral infection.^272^ The activated IRF3 dimer translocates into the nucleus, where it coordinately integrates with several transcription factors for promoters of IFN-I to trigger antiviral innate immunity. Zhang et al. recently reported that upon RNA virus infection, the JMJD6 recruits RNF5 from cytoplasm to the nucleus for degrading activated IRF3 via K48-linked poly-ubiquitination, maintaining the immune system homeostasis.^273^ Qin et al. indicated that during the late phase of viral infection, RNF26 negatively modulates the virus-triggered IFN-I production by indirectly impairing the IRF3 stability in an autophagy-dependent degradation manner.^262^ However, the relevant mechanism by which RNF26 mediates the autophagic degradation of IRF3 remains less known. Their findings indicated the temporal regulation of RNF26 on IFN-I production via distinct mechanisms, by which RNF26 exhibits a positive regulator in IFN-I responses in the early phase of viral infection while displaying as a negative modulator at the late stage. RNF55 (c-Cbl) directly interacts with the nuclear IRF3 for promoting K48-linked poly-ubiquitination and proteasomal degradation of IRF3, thereby downregulating TLR- and RLR- mediated induction of IFN-I.^274^ Their study, combined with Cao’s findings, revealed the dual inhibitory function of RNF55 in RLR-mediated IFN-I signaling for the termination of excessive immune responses,^211,274^ indicating that the RNF55 is one of the promising new therapeutic targets for controlling excessive immune responses-related diseases. Interestingly, Jia et al. have demonstrated that the RNF125 negatively regulates the stability of TRIM14, which recruits the NEMO via Ub chains to MAVS signalosome to activate both IRF3 and NF-κB via enhancing the K48-linked polyubiquitination and proteasomal degradation of TRIM14 upon RNA virus infection.^275^

### NF-κB

NF-κB is a crucial dimeric transcription factor in antiviral innate immune responses, whose predominant form consists of p50 (NF-κB1) and p65 (RelA) subunits.^276^ NF-κB is expressed in the cytoplasm of all cell types and remains inactive through associating with IκBα to inhibit its translocation to the nucleus.^277^ The NF-κB pathway is activated by signaling through multiple receptors, including TLRs, RLRs, and cGAS, leading to inflammatory cytokines and IFN-I.^278,279^ After viral infection, the IKK signalosome complex is activated by upstream adaptors or kinases. The culmination of phosphorylated IκBα by activated IKK leads to its ubiquitination and proteasomal degradation, which sets free NF-κB and allows its translocation into the nucleus to bind DNA, controlling the production of IFN-I and inflammatory cytokines.^280,281^ Accumulating studies have indicated that RNF proteins emerged as viral molecules in orchestrating the activation and stability of NF-κB (Figs. 2–4).

RNF114 interacts with and ubiquitinates Zinc finger protein A20, which removes the K63 poly-Ub from several NF-κB signaling intermediates to inhibit NF-κB activation and mediate the K48-linked poly-ubiquitination of multiple substrates for their degradation to suppress downstream signaling, to stabilize A20 for restraining NF-κB responses.^282,283^ Besides, RNF114 negatively regulates NF-κB signaling by ubiquitinating and stabilizing IκBα to inhibit NF-κB-dependent transcription.^282^ Upon HIV-1 infection, RNF115 (BCA2), upregulated by activated NF-κB signaling, also promotes the stability of IκBα via strengthening the SUMOylation of IκBα, which modification severely impaired the phosphorylation of IκBα to prevent its proteasomal degradation, thereby inhibiting NF-κB activity by blocking its nuclear translocation in a negative feedback manner.^284^ The RNF115-mediated negative feedback loop significantly suppresses the expression of NF-κB-responsive genes, such as *BCA2*, and the HIV-1 provirus, leading to repression of HIV-1 infection, which further supports that the RNF115 is one of the promising therapeutic targets for inhibiting HIV-1 infection and its related diseases. RNF116 (CHFR) is directly associated with and ubiquitinated Aurora-A for its degradation, negatively regulating NF-κB signaling via reducing the Aurora-A-mediated IκBα phosphorylation.^285,286^ Tomita et al. demonstrated that loss of RNF116 results in accumulation of Aurora-A to promote the NF-κB activity through potentiating the phosphorylation and degradation of IκBα, thereby enhancing the growth and survival of HTLV-1-infected T cells.^287^

Besides, during SeV infection, RNF19B (NKLAM), an ISG induced by SeV infection in mice, positively regulates the nuclear translocation and phosphorylation of NF-κB subunit p65, enhancing the transcriptional activity of NF-κB.^288^ The deficiency of *Nklam* would significantly impair the activity of STAT1 and NF-κB, reducing IFNs and inflammatory cytokines in the lung. However, the genome expression of SeV in the lung is lower in *Nklam*^-/-^ mice than in the wild type, and survival overall has no difference. The LUBAC, composed of SHARPIN, RNF54 (HOIL-1), and RNF31 (HOIP), can conjugate linear (M1-linked) poly-Ub chains onto NEMO/IKKγ to activate the canonical NF-κB pathway is downstream of both TNFRSF and RIG-I.^289–291^ A recent study has indicated that during IAV infection, the expression of RNF54 (HOIL-1) is significantly upregulated by a mechanism involving the direct binding of IRF1 to the RNF54 promoter in response to IFN-I.^291^ The upregulation of RNF54 can promote LUBAC formation to evoke an exaggerated inflammatory response. The in vivo evidence showed that the productions of IFN-I and inflammatory cytokines are reduced in mice with lung epithelial-specific deletions of HOIL-1 compared with wild type, while the anti-inflammatory cytokine IL-10 increased, thus reducing lung injury, improving survival in mice infected with IAV. This study suggested that upon IAV infection, the positive feedback loop, IAV-LUBAC (RNF54)-NF-κB-IFN-RNF54-NF-κB, exaggerates the production of cytokines, which may contribute to the morbidity and mortality observed in severe cases of influenza infection. Their findings provided a promising potential target at RNF54 for controlling IAV-induced cytokine storms during severe infection. RNF121, a Golgi apparatus-anchored E3 ubiquitin ligase, promotes the proteasomal degradation of IκBα to positively regulate the activation of the NF-κB signaling pathway following stimulation of TLRs and RLRs in a catalysis‐dependent manner.^292^ However, RNF121 does not directly ubiquitinate IκBα, and additional investigation is required to determine the molecular mechanism by which RNF121 facilitates IκBα degradation.

Conversely, several RNF proteins also inhibit the NF-κB activity or stability. RNF182, specifically upregulated by TLR stimuli, promotes the K48-linked poly-ubiquitination and proteasomal degradation of cytoplasmic p65, thus repressing TLR3-triggered proinflammatory responses.^293^ Their findings identified that RNF182 acts as a feedback-negative regulator of TLR-induced inflammation for restraining proinflammatory cytokine production from maintaining immune homeostasis by mediating the ubiquitination and degradation of p65. RNF216 (ZIN/Triad3A) directly interacts with receptor-interacting protein (RIP) and mediates the K48-linked poly-ubiquitination of RIP for proteasomal degradation, thereby dampening NF-κB activation.^294^ Therefore, RNF216 plays an important role in limiting TLR and RLR signaling intensity and duration to prevent overreacting host damage during virus infection, supporting RNF216 as a promising potential target to restrict viral infection or maintain the immune system homeostasis.^190,191,249^ RNF198 (RC3H1), an RNA-binding protein, directly binds to the 3′UTR of A20 mRNA via ROQ and CCCH-Znf domains to promote its degradation, thus positively modulating the NF-κB activity.^295^ This report highlighted that RNF198 acts as a novel *cis*-acting recognition element in the A20 3′UTR to control the expression of several NF-κB pathway regulators. This study provided a potential target at RNF198 for upregulating A20 to improve patient outcomes in certain disease scenarios.

### JAK-STAT signaling pathway and its downstream ISGs

**Fig. 5:**
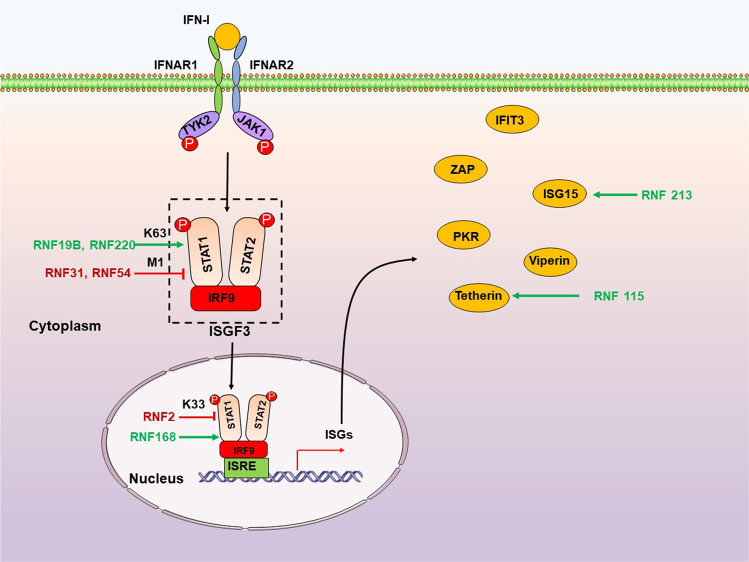
RNF proteins regulate the JAK-STAT signaling pathway and its downstream ISGs. The antiviral activities of IFN-I are initiated by binding to their cognate receptor IFNAR1 and IFNAR2 to trigger a signaling cascade, namely JAK-STAT pathways. The activated JAKs then trigger the assembly of the ISGF3 complex, which imports into the nucleus to bind ISRE for ISG production. RNF proteins are responsible for positively (green arrows) or negatively (red T-shaped solid line) modulating the JAK-STAT signaling pathway and its downstream ISGs. P, phosphate

The antiviral activities of IFN-I and inflammatory cytokines can be initiated by binding to their cognate receptor IFNAR1 and IFNAR2, which cytoplasmic domains interact with JAK tyrosine kinases, and the binding induces the formation of receptors to allow the trans-phosphorylation of tyrosine kinase 2 (Tyk2, one member of JAK tyrosine kinases) and JAK1.^296^ The activated JAKs subsequently lead to the phosphorylation and heterodimerization of STAT1/STAT2, which, together with the transcription factor IRF9, form a complex known as IFN-stimulated gene factor 3 (ISGF3) that import into the nucleus to combine the IFN stimulated response element (ISRE) for numerous ISGs production to battle the invading viruses.^297,298^ Several RNF proteins are responsible for modulating key molecules’ activity and stability of the JAK-STAT signaling pathway and its downstream ISGs (Fig. 5).

#### JAK-STAT signaling pathway

STAT1, a cytoplasmic transcription factor, is the only member of the STAT family that can be activated by all types of IFNs.^299^ The regulations on the IRNAR-JAK-STAT signaling pathway by RNF proteins predominantly focus on the activity and stability of STAT1, thereby modulating the transcription of ISGs for combating invading viruses (Fig. 5). The myeloid-cell-specific RNF2-deficient (*Rnf2*^fl/*fl*^*Lyz2*-Cre) mice are more resistant to VSV infection than *Rnf2*^fl/*fl*^ littermates for enhanced ISGs production.^300^ Under the stimulation of IFN-I, nuclear RNF2 (RING1B) directly binds to STAT1, thus increasing its K33-linked poly-ubiquitination at position K379 to separate STAT1/STAT2 from DNA. The RNF2-mediated PTM of STAT1 consequently suppresses transcription of ISGs and subsequent antiviral responses. Their findings revealed a novel function of K33-linked poly-ubiquitination in antiviral innate immunity. The RNF31 (HOIP) is constitutively associated with STAT1, and both RNF31 and RNF54 (HOIL-1) are involved in the linear (M1-linked) ubiquitination of STAT1 at Lys511 and Lys 652 for inhibiting the binding of STAT1 to IFNAR2 to block activation of IFN-STAT1 signaling.^301^ The in vivo evidence showed that knockdown of HOIL-1 in *Rbck1*^+/−^ mice, widely used as an effective linear ubiquitination-deficient model, significantly reduces the levels of STAT1 linear ubiquitination in the lung, spleen, liver, and heart, as well as enhances the STAT1 activation in the lung and expression of the representative ISGs in the different organs, resulting in reduced viral loads in the organs of *Rbck1*^+/−^ mice. In addition, with SeV and VSV infection, the mRNA and protein levels of HOIP are upregulated. The interaction between HOIP and STAT1 was also enhanced, thus promoting the STAT1 linear ubiquitination to suppress IFN-I antiviral activity.

In contrast, RNF proteins are also involved in modulating the STAT1 activity and stability to potentiate the activation of STAT-1-mediated signaling. During IFNγ stimulation, RNF19B (NKLAM) is transiently localized to the IFNγ receptor complex, where it physically interacts with STAT1 to promote the K63-linked poly-ubiquitination of STAT1, potentiating the transcriptional activity and DNA binding ability of STAT1.^302^ They also revealed that upon SeV infection, RNF19B (NKLAM) positively regulates the phosphorylation of STAT1 to enhance its transcriptional activity.^288^ Guo et al. recently indicated that virus/IFN-induced RNF220 directly associates with STAT1 and attaches K63-linked poly-Ub to STAT1 at residue Lys110, which modification enhances the interaction between STAT1 and JAK1, thereby promoting the phosphorylation and activation of STAT1.^303^ The deficiency of RNF220 enhances host susceptibility to *A. baumannii* and HSV-1 infection for impaired STAT1-dependent IFN-I and IFNγ signaling. Their studies identified that RNF220 acts as a feedback-positive regulator to prolong the IFN-triggered immune response by mediating the ubiquitination and activation of STAT1. Besides function on STAT1 activity, a recent report has demonstrated that in oesophageal cancer, the RNF168, located in the nuclear, has a high frequency of gene amplification, which is correlated with overall poor survival for oesophageal cancer patients.^304^ The knockdown of RNF168 dramatically inhibits the proliferation and invasion of oesophageal cancer cells. They further indicated that the RNF168 potentiates the activation of JAK-STAT signaling via directly interacting with STAT1 to inhibit its ubiquitination and degradation with unknown mechanisms, thus leading to reduced expression of JAK‐STAT target genes in cancer cells. Considering the oncogenic role of STAT1 in oesophageal cancer, the RNF168 may be a promising new therapeutic target for oesophageal cancer therapy.

#### ISGs.

##### Tetherin

Tetherin (also called BST2) is an ISG with a broad antiviral spectrum by restricting enveloped virus release from the cell surface as well as facilitating TLR3/7-mediated innate immune responses, activation and function of NK cell, CD4 T cell, and CD8 T cell, and antibody-dependent cellular cytotoxicity to control viral replication.^305^ Tetherin is a transmembrane protein that localizes to the plasma membrane, the trans-Golgi network, the early and recycling endosomes, and cycles between these membrane compartments.^306^ The topology of tetherin includes an N-terminal cytoplasmic domain followed by a single-pass transmembrane domain, an extracellular coiled-coil domain, and a C-terminal glycosylphosphatidylinositol anchor, which unique topology allows one end of the tetherin to be attached to the above membrane compartments and the other to the viral envelope.^307^ Retained virions are subsequently internalized for degradation via the endosomal pathway. RNF115 (BCA2) has been shown to directly associate with the cytosolic domain of tetherin to accelerate the internalization of viral particles captured by tetherin on the plasma membrane, leading to the lysosomal degradation of HIV-1 virions in an RNF115’s catalysis‐independent manner.^308^ Their data might provide therapeutic clues on RNF115 for controlling AIDS and related disorders. Further investigation is needed to address how RNF115 enhances the internalization of virions captured by tetherin and whether internalized viral particles can cycle back to the cell surface if not degraded.

##### ISG15

ISG15, a member of the family of ISGs, is the first identified ubiquitin-like protein with two ubiquitin-like domains with considerable homology to ubiquitin.^309^ Like ubiquitin, ISG15 conjugated via its C-terminus to substrate Lys residues, leading to its ISGylation, mediated by a ubiquitin-activating enzyme E1, UBE1L, a ubiquitin-binding enzyme E2, UBCH8, and three ubiquitin ligase E3s, HHARI, TRIM25, and HERC5. ISG15 and its conjugation machinery are strongly upregulated by IFN-I, IFN-III, and viral nucleic acids, thereby modulating the antiviral immunity by ISGylation of key molecules of immune signaling pathways and inhibiting viral replication through ISGylation of viral proteins. For instance, ISG15-mediated CARD ISGylation is required for MDA5 activation via facilitating the formation of higher-order MDA5 assemblies, thereby enhancing MDA5-mediated restriction of a range of RNA viruses.^310^ A recent study reported that RNF213 acts as a sensor for ISGylated proteins modified by ISG15 on lipid droplets upon IFN signaling to facilitate host immunity against virus.^311^ Besides, in vitro and in vivo evidence showed that RNF213 exhibits a broad antimicrobial activity, including *Listeria monocytogenes*, HSV-1, human respiratory syncytial virus, and coxsackievirus B3infection infection, which implicated that the RNF213 may be a promising therapeutic target for controlling various viral pathogens infection and their related diseases. Their findings revealed a novel mechanism by which ISG15 modification is recognized. However, additional study is required to determine what happens to ISGylated proteins after binding to RNF213.

### Adaptive immune responses

**Fig. 6:**
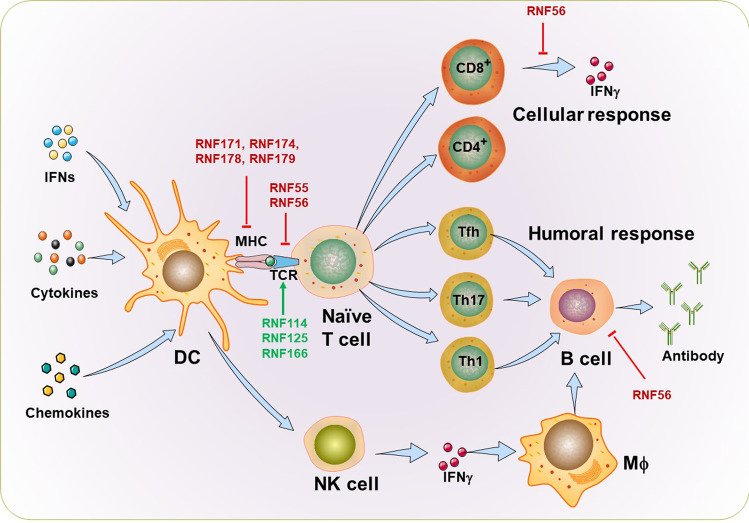
RNF proteins orchestrate the adaptive immune responses. After the innate immune responses, the DCs are activated by IFNs-induced cytokines and chemokines and then efficiently present the antigens to naive T cells via a combination of MHC and TCR. Naive T cells differentiate into several subtypes with unique functions in triggering and regulating T-cell-dependent cellular immune responses and B-cell-mediated antibody secretion

The activation of IFNs signaling drive the activation and maturation of antigen-presenting cells such as macrophages and dendritic cells (DCs), which subsequently present the antigens to naive T cell through a combination of major histocompatibility complex (MHC) and T cell receptor (TCR), thereby engaging the differentiation of naive T or B lymphocytes to initiate cellular and humoral immunity for eventual virus clearance.^312,313^ A few studies have identified that RNF proteins regulate several steps in adaptive immune responses against viral infection (Fig. 6).

Several studies have identified that the RING-UIM family members, including RNF114, RNF125, and RNF166, positively regulate T cell activation.^33,314–316^ These three members act as the positive regulators of TCR signaling-mediated T cell activation, using surface CD69 expression as a surrogate marker for cell activation. Silencing the RNF125 dramatically suppresses T cell activation in response to TCR cross-linking, and overexpression of RNF114 and RNF166 significantly upregulates the CD69 expression. However, additional studies are needed to address whether the RING-UIM proteins-mediated regulation of T cell activation plays a role in viral infection. In contrast, the MARCH family proteins exert an inhibitory effect on adaptive immunity via targeting the MHC molecules.^25,317^ RNF171 (MARCH 1) and RNF178 (MARCH 8) play pivotal regulatory roles in lymphocyte development. RNF171 and RNF178 target the MHC II HLA molecules for HLA internalization and lysosomal degradation to inhibit CD4 T cell activation.^318^ In addition, the MHC I ubiquitination by RNF174 (MARCH 4) and RNF179 (MARCH 9) enhances its internalization and degradation to suppress CD8 T cell activation.^319,320^ Further investigation is needed to address whether the MARCH members-mediated inhibition of T cell activation participates in viral restriction.

The RNF55 (c-Cbl) and RNF56 (Cbl-b) also function as negative regulators in T cell activation by promoting ligand-induced TCR down-modulation, consequently contributing to the termination of TCR signals.^321^ In addition, upon TCR stimulation, the RNF56 inhibits the Vav-1 activation and inactivates PTEN via attaching K63-linked ubiquitination at PTEN K13 to downregulate the T cell activation.^122,322,323^ Importantly, RNF56 plays a pivotal role in CD8^+^ T cell-dependent viral control.^324^ During acute lymphocytic choriomeningitis virus (LCMV) infection, the *Cbl-b* ablation constraints antigen-induced TCR down-modulation by effector CD8 T cells, leading to augmented IFNγ production, while does not affect the primary activation, clonal expansion, functional affinity, as well as cellular cytotoxicity of virus-specific CD8 T cells, thereby further supporting the crucial role for RNF56 in the termination of TCR signaling and protection against T cell-dependent autoimmunity.^122^ However, the viral loads are not significantly different in the spleens between P14/Cbl-b ^+/+^ and P14/Cbl-b^−/−^ CD8 T cell recipients, implicating the RNF56-dependent CD8 T cells responses may be useless in viral restriction. This study provides a comprehensive understanding of the regulation of CD8^+^ T cell responses by RNF56 after acute viral infection and implicates the potential therapies via altering CD8 T cell effector function. Besides, during VSV infection, deficiency of *Cbl-b* restores virus-specific IgG production, IgG class switching, and germinal center (GC) formation in Vav1 mutant mice, implicating the RNF56 may be involved in modulating CD40 signaling-mediated GC formation.^325^ Loss of *Cbl-b* dramatically enhances the CD40-induced B cell proliferation, antibody production, and GC formation, while CD40 ablation abrogates these effects.^326^ They further revealed that RNF56 interacts with TRAF2 upon CD40 ligation to inhibit the recruitment of TRAF2 to CD40, selectively impairing CD40-mediated activation of NF-κB and JNK.

## Concluding and future perspectives

In summary, RNF proteins exhibit a crucial role in health and disease for their widespread involvement in almost every cellular process. Particularly in cancer development and progression, the oncogenic role and overexpression of MDM2, caused by gene amplification, are observed in a variety of human tumors, as well as the genetic alterations, including mutations, genetic deletions, and promoter hypermethylation, of tumor-suppressive Parkin, RNF43, and RNF55 often occur in a range of cancers. Elucidating the underlying mechanisms of genetic alterations of RNF proteins in the pathophysiology of cancers and other diseases may provide insights into developing novel and more effective therapeutic strategies. The design of drugs that target the specific genetic alterations of RNF proteins to avoid their dysregulation could be one of the formidable challenges in the future.

The innate immune responses are the first line of host defense, characterized by the production of IFN-I and ISGs to restrict viral infection and transmission. Numerous studies have gradually unveiled that RNF proteins have played a crucial role in accurately orchestrating the key signaling molecules and their relevant pathways in the past decades. Our more recent review has briefly summarized the participation and mechanisms of RNF proteins in SARS-CoV-2 infection for developing novel effective vaccines, diagnoses, and therapies for COVID-19, which is currently the most formidable challenge to humans.^327^ However, the roles of other RNF proteins, except TRIMs, have not been fully addressed. Here, we have discussed the current knowledge about the RNF protein’s involvement in regulating the TLR, RLR, cGAS-STING, JAK-STAT pathway, and downstream ISGs. Substantial studies have demonstrated that the key signaling molecules are activated and degraded by various RNF proteins – mediated K27-linked, K33-linked, K48-linked, K63-linked, or M1-linked ubiquitination at same or different Lys residues, dynamically controlling the antiviral innate immunity against invading virus. Except for canonical K48 or K63 linkages, the function and characterization of atypical linkage types by RNF proteins require additional confirmatory investigations. Besides, the underlying mechanisms by which RNF proteins modulate innate antiviral signaling in a catalysis/ubiquitination‐independent manner are needed to unveil in greater detail. Furthermore, additional studies on the dynamic and spatial regulation of the same Lys residue of the same substrate by diverse canonical or atypical ubiquitination may provide new insights into proper immune defense.

This review elucidates the roles of RNF proteins, except TRIMs, in the pathogenesis of some diseases, including cancer, autoimmune diseases, a neurodegenerative disorder, and viral infection, and provides the potential intervention strategies targeting other RNF proteins. Although RING-type E3s represent promising new therapeutic targets for some human diseases, several factors impeded the clinical applications of RNF proteins for small molecule therapeutics. Gaining further insights into the crystal structures of RING-type E3s and their ligand-bound complexes would improve the structure-based design to develop specific small molecules targeting these RNF proteins, ultimately for therapeutic applications.

## Acknowledgements
